# First-in-human controlled inhalation of thin graphene oxide nanosheets to study acute cardiorespiratory responses

**DOI:** 10.1038/s41565-023-01572-3

**Published:** 2024-02-16

**Authors:** Jack P. M. Andrews, Shruti S. Joshi, Evangelos Tzolos, Maaz B. Syed, Hayley Cuthbert, Livia E. Crica, Neus Lozano, Emmanuel Okwelogu, Jennifer B. Raftis, Lorraine Bruce, Craig A. Poland, Rodger Duffin, Paul H. B. Fokkens, A. John F. Boere, Daan L. A. C. Leseman, Ian L. Megson, Phil D. Whitfield, Kerstin Ziegler, Seshu Tammireddy, Marilena Hadjidemetriou, Cyrill Bussy, Flemming R. Cassee, David E. Newby, Kostas Kostarelos, Mark R. Miller

**Affiliations:** 1 BHF Centre for Cardiovascular Science, University of Edinburgh, Edinburgh, UK; 2 The Royal Infirmary of Edinburgh, Edinburgh, UK; 3 Nanomedicine Lab, Faculty of Biology Medicine and Health, The University of Manchester, Manchester, UK; 4 National Graphene Institute, The University of Manchester, Manchester, UK; 5 Catalan Institute of Nanoscience and Nanotechnology (ICN2), CSIC and BIST, Campus UAB, Barcelona, Spain; 6 Centre for Inflammation Research, University of Edinburgh, Edinburgh, UK; 7 National Institute for Public Health and the Environment (RIVM), Bilthoven, The Netherlands; 8 Division of Biomedical Sciences, University of the Highlands and Islands, Inverness, UK; 9 Lydia Becker Institute of Immunology and Inflammation, The University of Manchester, Manchester, UK; 10 Thomas Ashton Institute for Risk and Regulatory Research, The University of Manchester, Manchester, UK; 11 Institute for Risk Assessment Sciences, Utrecht University, Utrecht, The Netherlands; 12 Institució Catalana de Recerca i Estudis Avançats (ICREA), Pg. Lluís Companys 23, Barcelona, Spain

**Keywords:** Nanoparticles, Nanostructures

## Abstract

Graphene oxide nanomaterials are being developed for wide-ranging applications but are associated with potential safety concerns for human health. We conducted a double-blind randomized controlled study to determine how the inhalation of graphene oxide nanosheets affects acute pulmonary and cardiovascular function. Small and ultrasmall graphene oxide nanosheets at a concentration of 200 μg m^−^^3^ or filtered air were inhaled for 2 h by 14 young healthy volunteers in repeated visits. Overall, graphene oxide nanosheet exposure was well tolerated with no adverse effects. Heart rate, blood pressure, lung function and inflammatory markers were unaffected irrespective of graphene oxide particle size. Highly enriched blood proteomics analysis revealed very few differential plasma proteins and thrombus formation was mildly increased in an ex vivo model of arterial injury. Overall, acute inhalation of highly purified and thin nanometre-sized graphene oxide nanosheets was not associated with overt detrimental effects in healthy humans. These findings demonstrate the feasibility of carefully controlled human exposures at a clinical setting for risk assessment of graphene oxide, and lay the foundations for investigating the effects of other two-dimensional nanomaterials in humans. Clinicaltrials.gov ref: NCT03659864.

## Abstract (web summary)

Assessment of the health risks of exposure to anthropogenic nanomaterials is crucial to maximize their potential applications. This double-blind, randomized controlled study in healthy humans evaluates the impact of inhalation of graphene oxide nanosheets on acute pulmonary and cardiovascular functions.

## Main

Two-dimensional (2D) nanomaterials are defined as flat, non-spherically shaped substances with a least one dimension <100 nm, and have generated worldwide interest for a variety of potential applications including building materials, car tyres, inks, food preservatives, sun-screens and anti-corrosion and lubricating products. Graphene is an archetypal 2D nanomaterial of a single layer or few layers of carbon lattice. Its unique structure, strength, flexibility, transparency and electrical conductance properties make it attractive for a wide range of applications^1^. There is also intense interest in further developing such materials for biomedical applications, including diagnostic and drug-delivery agents^2,3^. The oxidized form of graphene, graphene oxide (GO), has shown promise in the biomedical setting due to its hydrophilicity, high surface area for chemical functionalization, reasonable colloidal stability in biologically relevant solutions and compatibility with blood cells^4^. However, like other manufactured nanomaterials, the safety profile and limitations of GO on human exposure need to be determined before widespread use. There are limited and inconsistent toxicological data available for GO, often arising from differences among the many different sources of the material and their notable variability in dimensions and chemical properties that do not allow confident conclusions to be reached regarding its safety^5^. We have systematically synthesized GO nanosheets with high control and homogeneity of size and minimal trace metal and no endotoxin contaminations^6^ that do not exert the overt toxicity reported for many commercial sources of other graphene material types (such as graphene nanoplatelets)^7–9^. These highly purified GO materials have been thoroughly investigated using in vitro and in vivo models by different laboratories during the past decade^10,11^. A recent repeated and long-term pulmonary exposure study of these GO nanosheets in mice found that large (micrometre range) lateral size and high doses induced pulmonary inflammation (although substantially less than long, rigid carbon nanotubes) and more persistent granulomas^12^. Smaller (nanometre range) GO nanosheet exposures have demonstrated a transient inflammatory response that resolved rapidly post-exposure^12–14^.

Parallels can be drawn between manufactured nanomaterials and the small particulate matter in air pollution^15^. Particulate matter exposure has been linked to adverse health effects in almost every organ of the body^16^, although the respiratory and cardiovascular effects drive the substantial morbidity and mortality associated with particulate matter. Ultrafine (nano-sized) particulate matter is likely to contribute to these effects, given the high deposition in the alveoli of the lungs, the high reactive surface area for a given mass and the penetration to systemic organs^16^. While different classes of manufactured nanomaterial have distinct properties, there are commonalities with ultrafine particulate matter with respect to some physiochemical features and the pathways by which they induce toxicological effects, such as inflammation and oxidative stress^15^. Although there is an expanse of large-cohort epidemiological data linking particulate matter in air pollution with adverse effects, human data on the biological actions of manufactured nanomaterials are confined to cultures of human cell lines and biomonitoring in occupational settings or isolated accidental exposures in small groups of individuals^17^. Inhalation is the primary route of unintended pulmonary exposure to manufactured nanomaterials, but it also represents a promising route of administration for nanomedicines used for diagnosis and drug delivery for respiratory conditions. Therefore, human data are urgently needed to assess risk assessment and realize the true potential of these materials.

Controlled human exposure studies offer several advantages for assessing the acute biological effects of xenobiotics^17^. Unlike epidemiological studies, substances can be tested in isolation at defined doses. Real-world confounders, such as other environmental stressors (for example noise, stress, heat, exercise or medication) can be minimized or standardized across participants and study visits. The design can be tailored to include relevant control exposures, with repeated measure designs allowing each participant to be their own control. The use of a controlled exposure environment at clinics or laboratory facilities can broaden the range of endpoints to include a greater range of subclinical and mechanistic endpoints and has been used successfully to determine the respiratory and cardiovascular effects of combustion-derived nanoparticles such as those in diesel exhaust emissions^16,18,19^. However, only a handful of studies have tested the actions of manufactured nanomaterials, and no studies have been performed with non-spherical materials such as graphene^17^.

In this study we aimed to understand the potential for GO to have detrimental health effects, principally from the viewpoint of unintended exposure (for example occupationally or from public exposure with increasing use of nanomaterials in real-world applications) but also from the perspective of the development of safe forms of GO for intended human exposure by inhalation (for example for diagnostic imaging of the lung or drug delivery to or via the lung). Using a randomized controlled double-blind crossover design, we investigated the cardiorespiratory effects of acute inhalation of GO nanosheets in human volunteers. We hypothesized that inhalation of our high-purity, thin GO would have only modest effects on cardiorespiratory function and blood markers of inflammation and coagulability, the magnitude of which would be lower the smaller the lateral dimensions of the nanosheets. The findings of this controlled human inhalation exposure to GO in human volunteers lay the foundations for future investigations to establish which properties of graphene materials determine their biological actions to provide a formal risk assessment and allow safe-and-sustainable-by-design development for various applications.

## Synthesis and characterization of GO nanosheets

**Fig. 1:**
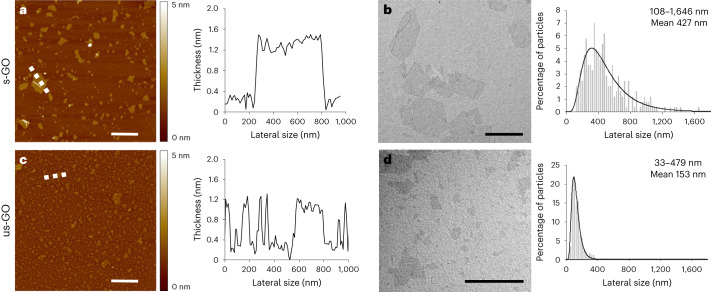
GO nanosheet size distributions. **a**–**d**, Morphological characterizations of s-GO (**a**,**b**) and us-GO (**c**,**d**). The images (left) and spectra (right) are representative of the technical replicates performed. Atomic force microscopy height images (left; **a** and **c**) with the corresponding cross-section analysis along the indicated dashed white lines (right; **a** and **c**). The colour bars indicate the height intensity range from 0 to 5 nm. Transmission electron microscopy micrographs (left; **b** and **d**) with the size distribution analyses using a Gaussian single peak fitting depicted with solid lines (right; **b** and **d**) are shown. Scale bars are 1 µm. The lateral size range and means given in **b** and **d** are an average of 242 and 224 individual GO sheets, respectively (see a summary table in Extended Data Table 1).

**Extended Data Fig. 1:**
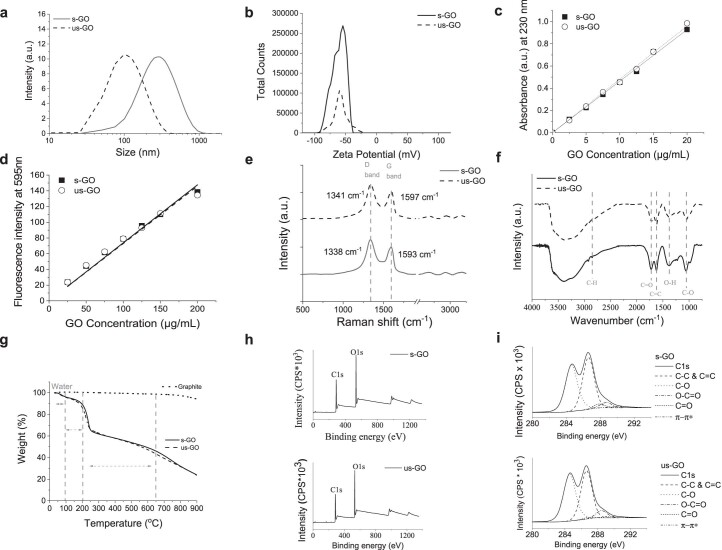
Physicochemical properties of s-GO and us-GO. **a**. Dynamic light scattering (DLS) confirms the size reduction from s-GO to us-GO; **b**. Electrophoretic light scattering (ELS) shows highly negatively charged particles, suggesting a high colloidal stability; **c**. Absorption spectroscopy confirms the C=C grapheneous backbone signature through its absorbance linearity at 230 nm; **d**. Upon excitation with λexc = 525 nm (fluorescence spectroscopy), a maximum band at λem ~ 595 nm is generated by electrons transitioning between the oxidized and non-oxidized carbon atoms of the GO sheets; **e**. Raman spectroscopy highlights a GO-specific spectral signature through D and G bands, an indicator of functional groups (defects) presence onto the grapheneous backbone; **f**. Fourier-transform infrared spectroscopy (FT-IR) confirms the presence of functional groups such as OH, CH, C=O; C=C and C-O; **g**. Thermal gravimetric analysis (TGA) also confirms the oxygenated nature of s-GO and us-GO, which show particular degradation steps that are associated with the disruption of COOH, C=O, OH, C-O-C, as opposed to graphite which is stable at temperatures below 600 °C; **h**. X-Ray photoelectron spectroscopy (XPS) for the survey spectra indicate highly-pure materials of rich oxygen content. **i**. X-Ray photoelectron spectroscopy (XPS) for the high-resolution C1s spectra mainly involved in C-O, C=O and O-C-O covalent bonds. a.u. = arbitrary units; CPS = counts per second; eV = electronvolt. Data are presented as mean values ± SEM. n-numbers. Representative data of the technical replicates performed are shown.

Thin, highly purified metal-free and endotoxin-free GO materials were synthesized using a modified Hummers’ method and comprehensively characterized (Fig. 1, Extended Data Table 1 and Extended Data Fig. 1)^6^. GO nanosheets were of tightly defined size, with no metallic or other elemental contamination, residues or indicators of bacterial contamination^10^. Two lateral dimensions (maintaining all other physicochemical characteristics almost identical) were selected for the study: small GO (s-GO) and ultrasmall GO (us-GO). Both types of nanosheet have demonstrated no acute or longitudinal adverse effects in our previous pre-clinical (rodent) studies^6^, contrary to ‘large’ GO sheets that were thus excluded from this work as a safety precaution.

GO nanosheets were aerosolized for exposure of volunteers through inhalation via a face mask with a target mass concentration of 200 μg m^−^^3^. The actual concentrations of s-GO and us-GO were 214 ± 23 μg m^−^^3^ and 224 ± 17 μg m^−^^3^, respectively (Extended Data Table 2) and were maintained at a constant level throughout the 2 h exposure (Supplementary Fig. 1a). As anticipated, the us-GO had a greater particle number than the s-GO for the same mass. The GO agglomerated to airborne sizes with a median value of 80–90 nm and a high size distribution homogeneity (Supplementary Fig. 1b). The levels of GO used here (200 μg m^−^^3^) were substantially higher than concentrations of graphene materials found in many workplaces that handle/process these materials (0.4–50 μg m^−^^3^)^20–22^ and are relevant to proposed occupational guidance for graphene nanoplatelets (212 μg m^−^^3^)^23^.

## Clinical effects of inhaled GO

**Fig. 2:**
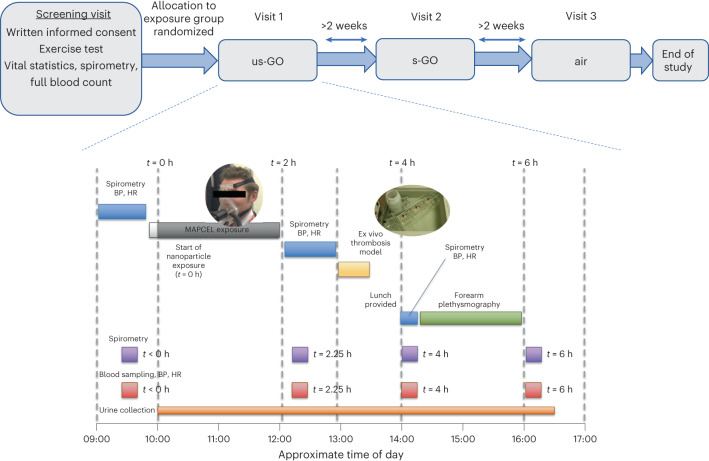
Study design and study day schedule. Top: timeline of randomized study visits. Bottom: study day schedule. Inset images show a volunteer inhaling exposures through a tubed face mask (left) and the ex vivo thrombosis chamber (right; see Fig. 4c). BP, blood pressure; HR, heart rate; MAPCEL, mobile ambient particle concentrator exposure laboratory.

This study was performed in accordance with the Declaration of Helsinki and rigorous ethical review at the University of Edinburgh and relevant UK National Health Service research and development office. Volunteers inhaled either GO or filtered air for 2 h under carefully controlled conditions while intermittently cycling (used to standardize respiratory rates between individuals based on exercise workload determined at the screening visit) (Fig. 2 and Supplementary Fig. 2). Of the 14 participants, 13 completed all 3 visits; one participant was unable to attend their third visit within the time frame of the study. All exposures were well tolerated, with participants reporting no symptoms related to exposure and only mild transient fatigue related to the exercise. No symptoms were reported throughout each study day, and there were no clinical adverse events of any description throughout the study. The lack of overt effects of acute inhalation of high-purity GO highlights the feasibility of controlled exposures of graphene-based nanomaterials for risk assessment in humans.

## Lung function

**Fig. 3:**
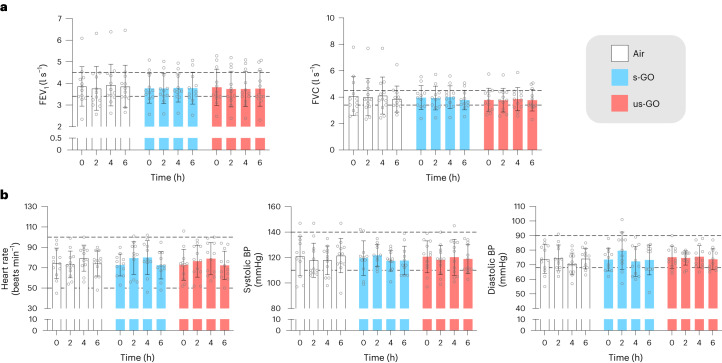
Lung function and cardiovascular vital signs for exposure to air, s-GO and us-GO. **a**, Lung function as determined by the forced expiratory volume in 1 s (FEV_1_, left) and forced vital capacity (FVC, right). **b**, Cardiovascular vital signs: heart rate (left), systolic BP (middle) and diastolic BP (right). The dotted lines represent the upper and lower limits of expected normal values and the coloured bars and error bars show the mean ± s.d. (*n* = 12 for air and us-GO groups, *n* = 11 for s-GO groups; biologically independent measurements). No significant differences were found between treatments (two-way analysis of variance followed by Tukey’s post hoc test). Source data

Spirometry is widely used to monitor changes in, or sensitization to, airway reactivity to assess the risk and progression of respiratory conditions such as asthma and chronic obstructive pulmonary disease. The forced expiratory volume in 1 s and forced vital capacity varied little across the time points within each exposure, and there were no differences in either of these parameters between different exposures (*P* > 0.42 for all comparisons; Fig. 3 and Extended Data Table 3). The findings demonstrate that the GO nanosheet exposures did not directly alter lung function after acute exposure. While studies in animal models have not found effects of GO on airway reactivity, GO has been found to induce pulmonary inflammation (see the Supplementary Information for a discussion). Biomarkers of pulmonary inflammation would be a valuable addition to future human-controlled nanomaterial exposures. Toxicological studies in mice with the same GO materials as the current study found that pulmonary exposure induced a mild and transient pulmonary inflammation^12–14^. It is possible that GO with greater lateral dimensions could have induced greater inflammation and changes to lung function, or that effects would have been observed in susceptible individuals, such as those with asthma.

## Systemic haemodynamic effects

Heart rate and blood pressure were measured at rest at the same time points as lung function. There were no differences in heart rate (*P* > 0.88) and systolic (*P* > 0.85) or diastolic (*P* > 0.5) blood pressure (Fig. 3 and Extended Data Table 3). The lack of effect was anticipated, as our previous volunteer studies did not find consistent increases in heart rate or blood pressure after acute exposure to diesel exhaust nanoparticles^19,24,25^. Only one study has investigated the effects of graphene material on blood pressure^26^, finding that dextran-coated graphene nanoplatelets had no effect on blood pressure in mice after high-dose (up to 250 mg kg^−1^) intravenous exposure. Many manufactured nanomaterials have oxidative properties that stimulate cellular and tissue oxidative stress. In small arterioles, oxidative stress would be expected to increase blood pressure via constriction of blood vessels and impairment of vasodilatory capacity. The lack of effect of GO on blood pressure and the absence of substantive changes in plasma isoprostanes following GO exposure in preliminary lipidomic analyses (see below) suggest that there was no induction of systemic oxidative stress in this study. This is supported by studies using cultured vascular endothelial cells that have demonstrated that while GO is internalized by the cells, it did not generate intracellular superoxide^27^. Nonetheless, further exploration of these mechanisms is warranted in future human studies, especially for the assessment of the risk of emerging graphene materials with different redox properties.

## Blood coagulability

**Fig. 4:**
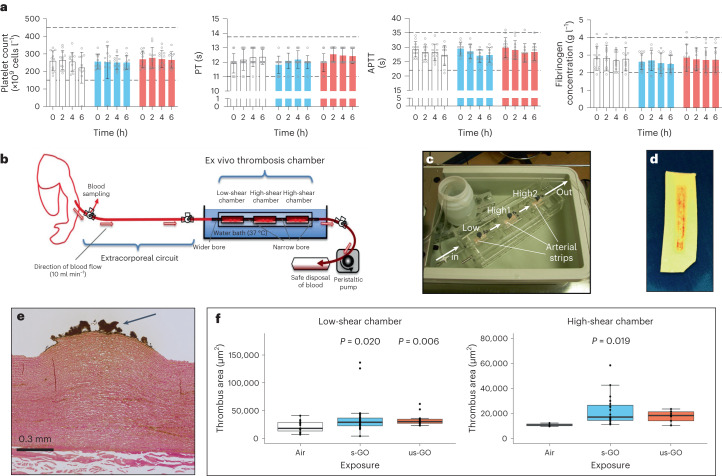
Blood coagulability parameters. **a**, Platelet counts and coagulability markers for exposure to air (white); s-GO (blue); or us-GO (red). The dotted lines represent the expected upper and lower limits of normal values. The colour bars and error bars show the mean ± s.d. (*n* = 12 for air and us-GO groups, *n* = 11 for s-GO groups; biologically independent measurements). No significant differences were found between treatments (two-way analysis of variance followed by Tukey’s post hoc test). **b**, Schematic of the ex vivo thrombosis chamber set-up for measuring blood thrombogenicity. **c**, Photograph of the chamber containing the three strips of porcine artery (pink) with the direction of blood flow across the chamber marked. Low, low-shear chamber; High1, first high-shear chamber; High2, second high-shear chamber. **d**, Porcine strip (white) taken from the chamber with adhering blood (vertical red line) seen running down the centre of the strip. A corner of the strip is removed to identify the direction of blood flow. **e**, A representative histological section of the strip (pink) with blood (brown, arrow) adhering to the intimal surface. **f**, Inhalation of either s-GO (*P* = 0.02) or us-GO (*P* = 0.006) led to a greater blood thrombogenicity compared with air exposure in the low-shear chamber. A similar pattern was observed in the high-shear chamber, although the effect was only demonstrable for s-GO (*P* = 0.019; for us-GO, *P* = 0.07). There was no statistically significant difference in thrombogenicity between s-GO and us-GO. The central lines of the box plots show the median, box ranges show the 25–75th percentiles and whiskers show the minima (25th percentile − 1.5 × interquartile range) and maxima (75th percentile + 1.5 × interquartile range); each point represents a single section from the arterial strip. Biologically independent measurements are from 13 volunteers (low-shear chamber) and 8 volunteers (high-shear chamber). Comparisons with air were made by one-tailed Kruskal–Wallis tests. APTT, activated partial thromboplastin time; PT, prothrombin time. Source data

An increased propensity of the blood to clot increases the risk of thrombosis within a blood vessel and concomitant cardiovascular events, such as a heart attack or ischaemic stroke. Human exposure to particulate matter in ambient air pollution is associated with raised markers of blood coagulation, such as fibrinogen, platelet activity and in vitro coagulation assays, although the data are inconsistent^28^. In this study, blood platelet counts and activated partial thromboplastin times were similar within each exposure (Fig. 4a and Extended Data Table 4). Although a marginal increase was observed in the prothrombin time after exposure (*t* = 2 h) in the us-GO group (*P* = 0.02), this increase was transient and remained within normal limits. There were no differences between exposures for any of these parameters. Blood fibrinogen, an essential glycoprotein of the coagulation cascade that is also an acute-phase response factor, was unchanged between exposures. See the Supplementary Information for further discussion.

One limitation of individual biomarkers and in vitro coagulation assays is that they overlook the interaction of blood with the vessel wall, an essential aspect of thrombosis. For this, we employed an ex vivo model of arterial thrombosis incorporating denuded porcine arterial strips, with different chambers that have flow conditions representing patent (low-shear chamber) and stenosed (high-shear chamber) human coronary arteries. Our previous studies in healthy volunteers demonstrated that acute exposure to diesel exhaust nanoparticles promoted the thrombogenicity of the blood in the absence of changes in individual biomarkers of coagulation^29,30^. In this study, inhalation of either s-GO (*P* = 0.02) or us-GO (*P* = 0.006) led to a greater blood thrombogenicity compared with air exposure in the low-shear chamber (Fig. 4b–f). A similar pattern was observed in the high-shear chamber, although the effect was only demonstrable for s-GO (*P* = 0.019; for us-GO, *P* = 0.07). No differences were observed when directly comparing s-GO with us-GO. The findings highlight that traditional coagulation markers are crude indicators of thrombogenicity, and that the inclusion of physiological conditions, such as the injured vessel wall, should be used to address the potential for thrombosis in vivo. The magnitude of the greater thrombogenicity after GO exposure was relatively mild and would be potentially of limited consequence, even in patients with pre-existing heart disease. Nevertheless, we recommend that characterization of the thrombogenic potential is included in experimental models used to develop risk assessments for graphene materials that may be of higher risk, or to consider higher exposure scenarios.

## Systemic inflammation

**Fig. 5:**
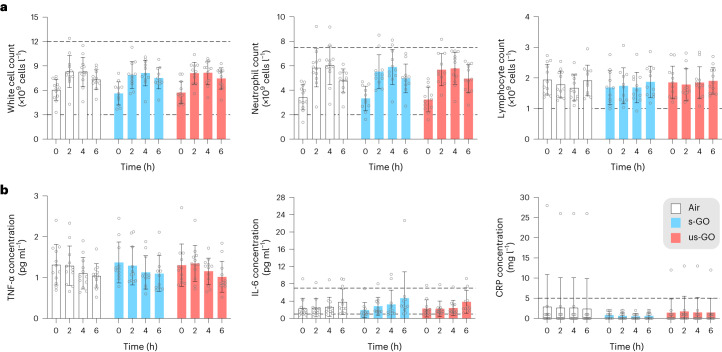
White blood cell counts and markers of inflammation. **a**,**b**, Inflammatory cell counts (**a**) and inflammatory cytokine concentrations (**b**) for exposure to air, s-GO and us-GO. The dotted lines represent the expected upper and lower limits of normal values. The coloured bars and error bars show mean ± s.d. (*n* = 12 for air and us-GO groups, *n* = 11 for s-GO groups; biologically independent measurements). No significant differences were found between treatments (two-way analysis of variance followed by Tukey’s post hoc test). Source data

Inflammation is a key mechanism underlying the cardiovascular effects of particles in air pollution in humans^31^ and various carbon-manufactured nanomaterials in animal models^32^. While white blood cell and neutrophil counts increased over the exposure period (*P* < 0.001 for all groups), the effect was identical in all the exposure groups, including filtered air (Fig. 5a and Extended Data Table 5). The lack of impact of GO in white blood cell counts aligns with those of a murine study of graphene nanoplatelet exposure^26^. There were no changes in the concentrations of the inflammatory cytokines: tumour necrosis factor alpha (TNF-α) or interleukin-6 (IL-6) over time or between different exposures (Fig. 5b and Extended Data Table 5). Minor differences in the baseline serum concentrations of the acute response protein C-reactive protein (CRP) were observed between exposure groups, but there were no changes between exposures when expressed as a percentage change from the baseline (*P* = 0.08 for s-GO versus air and *P* = 0.45 for us-GO versus air). The results suggest that physical exercise during the exposure increased the mobilization of white blood cells in the systemic circulation^33^. The exposure itself did not influence inflammatory cell number or activation.

Controlled exposure to diesel exhaust emissions can be associated with increased in markers of systemic inflammation in humans^18,25,34^, whereas exposure to spark-generated carbon black particles did not induce a systemic inflammatory response in healthy volunteers^29^. This is also in keeping with the available pre-clinical evidence: inhalation of GO in mice induced only mild increases in circulatory inflammatory cells or cytokines^9,35^ at high doses (>3 mg m^−^^3^) that are likely to be associated with lung overload and do not extrapolate to anticipated real-life exposure scenarios in humans^20,36^. The lack of effect of GO on markers of inflammation and the acute-phase response may reflect the purity of the GO, although the profile of the response at later time points remains to be confirmed (see the Supplementary Information for further discussion). Studies that make a direct comparison of our high-purity materials and commercial sources of GO that are typically less pure and more heterogenous in their size distribution would be valuable.

## Blood proteomic and lipidomic profiles

**Fig. 6:**
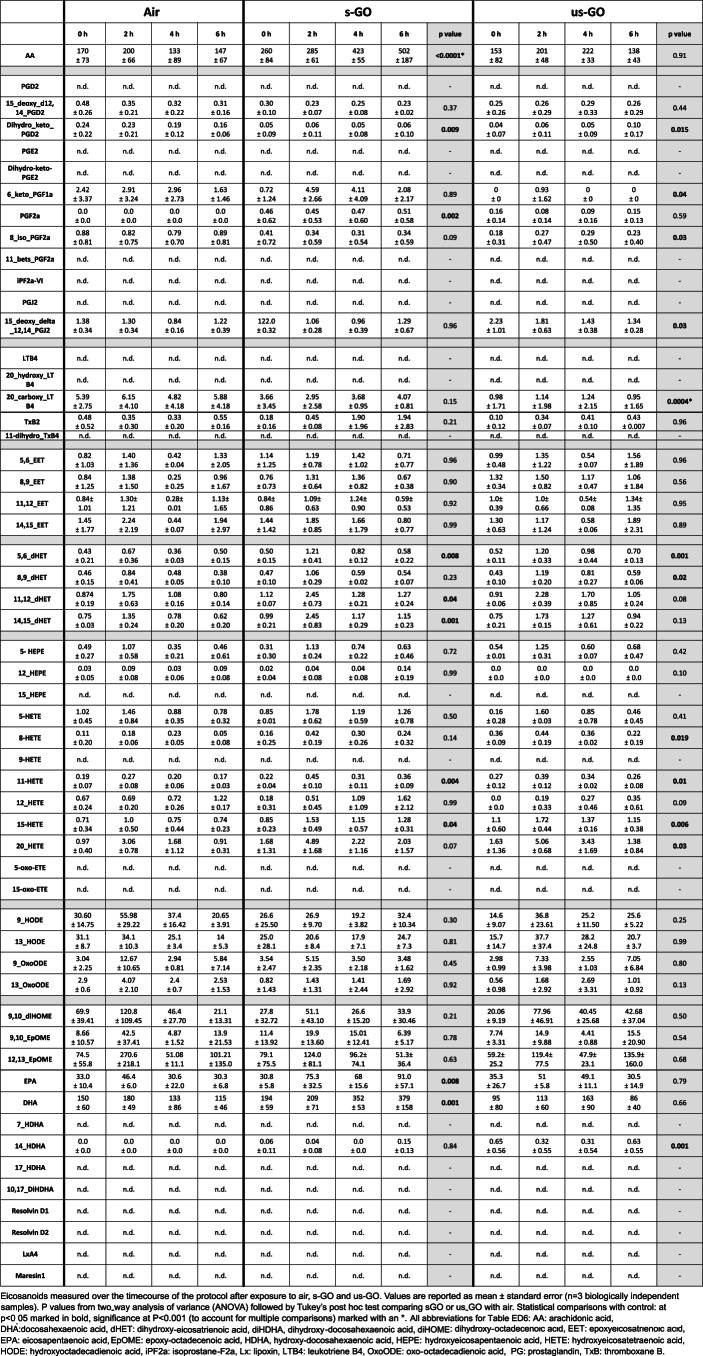
Blood proteome comparison from plasma samples using the NanoOmics enrichment pipeline. **a**, Heat map of normalized abundance values of all corona proteins identified by LC-MS/MS in samples collected before and after exposure to s-GO (left) and us-GO (right). A total of 692 (772) corona proteins were identified in s-GO (us-GO) exposure samples. Protein columns are sorted according to the abundance values (from highest to lowest) of the first sample. **b**, Volcano plots displaying the differential abundance of significantly different proteins before and after exposure to s-GO and us-GO (top) and between GO exposure groups and air at 6 h (bottom). Blue data points represent downregulated proteins and red data point upregulated proteins. The *n* values shown are from biologically independent samples. Filled dots represent the *n* = 1 common differentially abundant protein (SRI) between the two time points. The peptide intensities were compared between groups by one-way analysis of variance (ANOVA). Only proteins with *P* < 0.05 are shown. Source data

High-fidelity blood proteomics was used to probe for unanticipated acute systemic effects and further explore underlying pathways. A previously developed plasma enrichment approach (NanoOmics pipeline^37^) was employed before liquid chromotography tandem mass spectrometry analysis to minimize the masking effects from highly abundant plasma proteins (such as albumin and immunoglobulins) and to increase the dynamic range of the plasma proteomics data detected^38^. Lipid-based nanoparticles were used to adsorb proteins from the extracted plasma sample, forming a protein corona that can be recovered and purified from unbound proteins. Plasma from the baseline (*t* = 0 h) and end of the protocol (*t* = 6 h) were chosen for analysis, as the later time point was deemed the most likely to coincide with any inflammatory response to the GO exposure. A total number of 692 proteins were identified in the plasma of s-GO-exposed volunteers, of which only 3 were found to be differentially abundant (1 downregulated, 2 upregulated; Fig. 6) compared with the baseline (*t* = 0 h).

Similarly, out of 772 proteins identified in the plasma of us-GO-exposed volunteers, the abundance of only 4 proteins was shown to be affected (all upregulated) versus the baseline (*t* = 0 h). The proteomic analysis (Table 1) revealed that sorcin protein was upregulated after exposure to both s-GO and us-GO. Although previous studies have not associated sorcin protein with nanoparticle exposure, inflammation or toxicity, other studies have implicated sorcin in neurodegenerative diseases^39^ and calcium signalling in heart tissues^40^. Comparing between exposure groups, a total of 10 and 7 differentially abundant proteins were identified between s-GO and air exposures and between us-GO and air exposures, respectively. Of all the differentially abundant proteins, factor XII (air versus us-GO exposure groups) was the only protein identified that had immediate relevance to cardiorespiratory parameters of particulates, being implicated in both the complement and coagulation pathways^41,42^. In general, unlike previous diesel exhaust particulate exposure studies that have shown distinct plasma protein changes^43^, our analysis revealed only subtle changes in a very small number of plasma proteins.

**Table 1:** Identification of differentially abundant proteins from nano-enriched proteomic analysis of plasma samples

Description	Gene name	*P* value	Maximum fold change
s-GO: 0 h versus 6 **h (*n*** **= 3)**			
**Downregulated**			
cDNA FLJ26936 fis, clone RCT06808		0.019	5.52
**Upregulated**			
Platelet endothelial cell adhesion molecule	*PECAM1*	0.016	2.05
Sorcin	*SRI*	0.043	15.07
us-GO: 0 h versus 6 **h (*n*** **= 3)**			
**Upregulated**			
Fibroblast growth factor-binding protein 2	*FGFBP2*	0.024	1.91
Annexin		0.027	2.55
Proteoglycan 1, secretory granule, isoform CRA_a	*PRG1*	0.041	4.14
Sorcin	*SRI*	0.046	21.16
Air versus s-GO (6 **h) (*n*** **= 10)**			
**Downregulated**			
Tubulin alpha-4A chain	*TUBA4A*	0.007	3.99
Immunoglobulin G heavy chain		0.015	20.71
Prolactin-inducible protein	*PIP*	0.017	2.22
Alpha-1-antichymotrypsin (fragment)	*SERPINA3*	0.036	2.76
HRV Fab 026-VL (fragment)		0.042	1.53
60S ribosomal protein L18 (fragment)	*RPL18*	0.045	515.74
**Upregulated**			
Uncharacterized protein DKFZp686N02209	*DKFZp686N02209*	0.031	1.36
Phosphatidylinositol 3,4,5-trisphosphate 5-phosphatase 1	*INPP5D*	0.031	1.83
Collagen alpha-1(IV) chain	*COL4A1*	0.036	1.74
Putative ciliary rootlet coiled-coil protein 2	*CROCC2*	0.040	1.48
Air versus us-GO (6 **h) (*n*** **= 7)**			
**Downregulated**			
Coagulation factor XII	*F12*	0.006	5.27
Apolipoprotein B protein (fragment)	*APOB*	0.006	3.87
Plasma protease C1 inhibitor	*SERPING1*	0.02	1.46
Alpha-2-HS-glycoprotein	*AHSG*	0.02	1.66
cDNA FLJ41054 fis, clone STOMA1000189		0.03	9.00
cDNA FLJ90052 fis, clone HEMBA1002767, highly similar to Beta-1,4-galactosyltransferase 2 (EC 2.4.1.-)		0.049	1.70
Immunoglobulin heavy chain variable region (fragment)		0.049	18.05

The listed values are from independent biological samples for each group (from top to bottom table: n = 3, n = 4, n = 10, n = 7). Data were filtered in Pyrogenesis LC-MC to present a 1% false discovery rate. Comparisons between groups conducted using one-way analysis of variance, with only proteins demonstrating *P* < 0.05 shown. cDNA, copyDNA.

Source data

**Extended Data Fig. 2:**
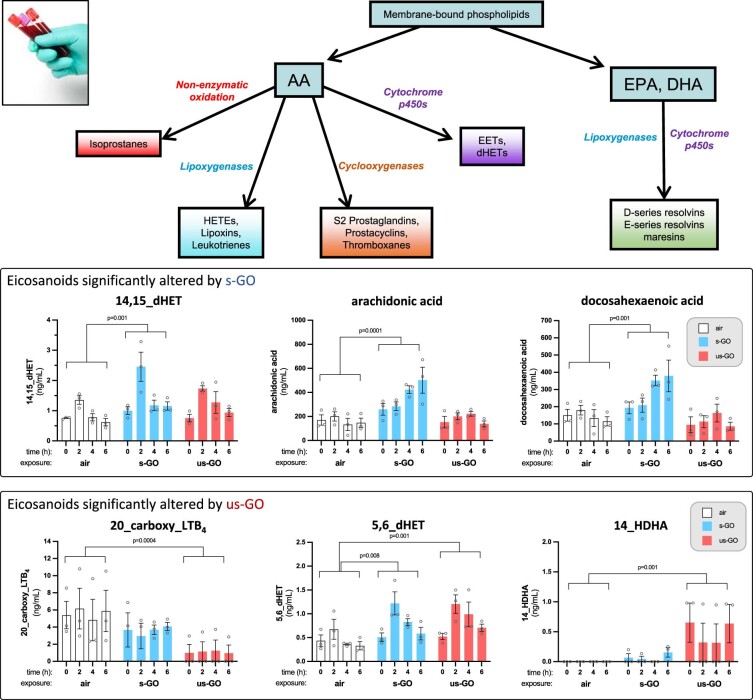
Eicosanoid measurements in blood. Eicosanoids measured over the time course of the protocol after exposure to air (white), small graphene oxide (s-GO, blue) and ultra-small graphene oxide (us-GO, red). Values are reported as mean ± standard error (n=3 biologically independent samples). dHET: dihydroxy-eicosatrienoic acid, EET: epoxyeicosatrienoic acid, EPA: eicosapentaenoic acid, HDHA: hydroxy-docosahexaenoic acid, HETE: hydroxyeicosatetraenoic acid, LTB4: leukotriene B4, PG: prostaglandin. Significance compared to air control, *P<0.05, **P<0.01, ***P<0.001. To account for multiple testing, *P*≤0.001 taken as statistically significant. Source data

Lastly, using lipidomic analysis of the extracted plasma samples we profiled eicosanoid species that may represent a means to detect more subtle changes in biological function that precede inflammatory biomarkers or functional changes in organ systems^44^. A preliminary exploration of plasma eicosanoids using targeted lipidomics found limited differences between some factors across the exposure groups (Extended Data Fig. 2 and Extended Data Table 6). Overall, the proteomic and lipidomic analyses corroborated the limited and mild effects of GO exposure on cardiorespiratory parameters and inflammatory mediators.

## Study limitations

While the use of controlled exposures in human participants has many advantages^17^, we acknowledge that our study has several limitations. First, the number of participants for this foundational study was only powered to detect changes in biological parameters based on our previous work with diesel exhaust nanoparticles. However, participant numbers may be insufficient to detect more subtle effects of GO inhalation. Second, we were only able to test a single dose of GO. For safety reasons, the dose was carefully chosen to avoid overt physiological effects, and it is possible that higher concentrations or longer durations of GO exposure could have actions that were not apparent in the current study. However, we note that our previous exposures to diesel exhaust used similar concentrations (100–300 μg m^−^^3^) of particulates and were accompanied by cardiovascular dysfunction measured using similar parameters^19,25,29^. Lastly, human-controlled exposure studies are limited to the exploration of acute exposure and restricted measurement periods. The study protocol could not be extended to more than 6 h after the start of the exposure and, therefore, slow-onset responses, such as some inflammatory pathways, would not have been captured.

## Conclusions

We found that the two GO sheets (s-GO and us-GO) with nanometre-sized lateral dimensions, minute thickness (1–2 nm) and high purity (in terms of both metallic and endotoxin contaminants) were largely innocuous in healthy volunteers at the dose and duration tested. This GO material was neither associated with acute changes in respiratory or cardiovascular function, nor systemic inflammation. While there was no major effect on blood coagulability, there was a mild increase in thrombogenicity in an ex vivo model of vascular injury, highlighting the need for comprehensive and subtle profiling of cardiovascular parameters to assess the actions of inhaled manufactured nanomaterials fully. This study lays the foundation for subsequent human studies investigating GO in larger numbers of individuals that could include differences in the degree of oxidation of GO (and surface oxygen content), purity and doses, as well as additional health parameters, time points and exploration of potential susceptible groups such as individuals with asthma and those with a greater risk of blood clotting. Great care should be taken to avoid generalizations from these results as the structural and surface characteristics of the GO materials and their purity, along with extensive pre-clinical investigations and knowledge, allowed the safe and ethical translation into the human exposures undertaken here. These studies could constitute a major advancement towards a comprehensive risk assessment of graphene and 2D nanomaterials to adopt a safe-by-design approach to harness the true potential of this unique material.

## Methods

See the Supplementary Information for full details.

### GO synthesis

Aqueous dispersions of s-GO and us-GO were prepared as described in our previous studies^6,45^ by a modified Hummers’ method coupled with sonication. We used depyrogenized glassware, handled under endotoxin-free conditions. Graphite powder was mixed with sodium nitrate and sulfuric acid by rigorous stirring at low temperature, followed by potassium permanganate and dropwise addition of water for injections. The mixture was stirred for 30 min at 98 °C before the reaction was stopped with hydrogen peroxide. Water for injections was used to neutralize the pH, remove impurities and separate the GO from the graphitic residues. GO was exfoliated by vortexing, and solubilized with warm water for injections from the orange gel layer. Any graphitic residues still present in the dispersion were removed by an additional centrifugation step 24 h post-reaction. Size reduction to small and ultrasmall flakes was carried out by sonication for 5 min and 4 h, respectively.

### Characterization of GO nanosheets

GO was comprehensively characterized by atomic force microscopy, transmission electron microscopy, hydrodynamic diameter and surface charge (zeta potential) measurements, UV–visible spectroscopy, fluorescence spectroscopy, Raman spectroscopy, Fourier transform infrared spectroscopy, thermogravimetric analysis and X-ray photoelectron spectroscopy (see the Supplementary Information for full details).

### Nanoparticle exposure and characterization

Nanoparticles exposures were performed in a mobile exposure laboratory positioned outside the Royal Infirmary of Edinburgh (Supplementary Fig. 2), under the supervision of an experienced exposure technician. Stock suspensions of GO (2 mg ml^−1^ for s-GO; 1.3 mg ml^−1^ for us-GO) were made in sterile distilled water that was free of any bacterial contamination, confirmed using a previously published method^10^. s-GO was diluted to 1.3 mg/ ml^−1^ in sterile saline in aseptic conditions, aliquoted and stored at 4 °C until use.

GO was transferred to a 5 ml syringe, placed on the syringe pump and aerosolized using a Schlick compressed air nebulizer (model 970/S Untersiemau, Dusen-Schlick), with in-line diluted with HPLC-grade water. The compressed pre-heated (60 °C) airflow of the Schlick nebulizer was 12 l m^−1^. The aerosol was dried in a heated mixing glass tube, then diluted with HEPA-filtered room air to the desired concentration and humidified to 50–60% relative humidity using an ultrasonic nebulizer (Omron Ultrasonic Nebulizer NE-U12). The aerosol was fed into a 200 l mixing chamber and delivered to the volunteer by an exposure mask placed over the mouth and nose, under a constant temperature and relative humidity (50%). GO was delivered at exposure concentrations between 100 and 300 μg m^−3^, with a target average concentration of 200 μg m^−3^. This dose range was chosen based on our previous controlled exposure studies with dilute diesel exhaust, which were associated with impairment of a range of cardiovascular parameters without adverse effects^19,30^ and with carbon and gold nanoparticles that did not alter cardiovascular parameters^29,46^. The concentration could be adjusted by altering the speed of the syringe pump delivering the suspension based on tapered element oscillating microbalance (model 1400 A, Thermo Scientific) readings, monitored and maintained by the exposure technicians.

The particle concentration in the aerosol was measured in the middle of the 200 l mixing chamber by an stainless steel tube. The particle characteristics measured were: particle mass (tapered element oscillating microbalance, as well as by gravimetric filter based analyses), particle number (condensation particle counter, model 3022 A CPC, TSI Inc.) and particle size distribution (PALAS differential electrical mobility classifier (U-DEMC model 2200) and an optical particle sizer (model 3330,TSI Inc.)). The particle mass was also determined post-exposure by calculating the accumulated mass on pre-weighed telfon filters taken from the metal tubing close to the volunteer exposure mask.

### Ethics statement

This study was designed with rigorous ethical review, with procedures being run by experienced clinicians and nursing support, and performed at a major hospital with the necessary emergency facilities should an adverse event have occurred. The study was performed in accordance with the Declaration of Helsinki, favourable ethical opinion of the University of Edinburgh, NHS Academic and Clinical Central Office for Research and Development (ACCORD), Research Ethics Committee (18-HV-084) and with written informed consent from all participants. The study has been registered on Clinicaltrials.gov under reference number NCT03659864.

### Participants and eligibility criteria

Fifteen healthy volunteers were recruited by advertising the study by posters and e-mails in the hospital and university campus, as approved by local ethical review. The data from 14 participants were included as one participant was unable to complete the exposure visits in the time frame of the study. The target of 15 individuals was chosen according to our previous controlled exposure studies with air pollutants based on changes to vascular reactivity and inflammatory cytokines in the blood from diesel exhaust exposure due to a lack of other controlled exposure studies of a 2D material for comparisons. A 1 h exposure to diluted diesel exhaust produced an ~32% reduction in forearm blood flow to 1 nmol min^−1^ bradykinin (~16 ± 2 versus ~19 ± 2.5 ml per 100 ml tissue per min (±s.d.) for diesel exhaust versus filtered air control, respectively^19^. A 2 h exposure to diluted diesel exhaust produced a 12.5% increase in plasma TNF-α (0.99 ±;0.07 versus 0.88 ± 0.007 pg ml^−1^ (±s.d.) for diesel exhaust versus filtered air, respectively^25^. On the basis of these figures, 12 and 10 volunteers, respectively, would be needed to detect these changes with significance of *P* < 0.05 with an 80% power. As other studies have not tested the effects of an inhaled 2D material, as an additional precautionary step, the decision was taken to not increase group sizes beyond 15 for this initial study.

Interested participants were provided with a participant information sheet that they were asked to read and consider for at least 24 h before agreeing to be involved in the study. For study visits, participants abstained from alcohol for 24 h and from food and caffeine containing beverages for at least 12 h before the study visit. Participants were invited for an initial screening visit to ensure that they met the inclusion criteria (Supplementary Table 2). The participants were asked about their occupation on their screening visit to rule out obvious exposures to particulates. We did not ask participants to wear a face masks outside of the study visits, as low compliance would have added an additional source of variability between participants (the study was run before the coronavirus pandemic, before mask wearing became common in the United Kingdom), and even occupational face masks have been shown to vary greatly in their removal of inhaled particles during different modes of activity^47^. Importantly, each volunteer acted as their own control and received each exposure in a random order, minimizing variation from both intrinsic biology and lifestyle factors.

### Study design

See Fig. 2. A screening visit was used to confirm eligibility criteria with the participant, followed by taking written consent and assignment of a participant code. Height, weight, heart rate, blood pressure and lung function were measured, and a 3 ml blood sample was taken for a full blood cell count. If parameters were within the normal range for young healthy individuals, participants were taken forwards to full study days. Participants also took a graded cardiorespiratory exercise stress test on a bicycle ergonometer to determine the workload required to generate a ventilation rate of 25 l min^−1^ m^−2^.

Two lateral dimensions of GO (maintaining all other physicochemical characteristics almost identical) were selected for the study: s-GO and us-GO. Both types of nanosheet have demonstrated no acute or longitudinal adverse effects in our previous pre-clinical (rodent) studies^6^, contrary to ‘large’ GO sheets that were thus excluded from this work as a safety precaution. A double-blind randomized crossover study design was used for the study visits, whereby the order of exposures (filtered air, s-GO, us-GO) were randomized. All study visits were organized at least 2 weeks apart to allow a wash-out period between different exposures. The volunteer and clinician performing the study were blinded to the identity of the exposure group. All researchers involved with collating and analysing the raw data were blinded to the exposure group, with unblinding occurring only when ready for grouping by exposure.

Before exposures (time *t* = 0 h), heart rate, blood pressure and lung function were measured, and blood taken. Participants were asked to empty their bladders and then given a urine container to collect any urine over the course of the study visit. Participants were then taken to the exposure laboratory based at the Royal Infirmary of Edinburgh site for the duration of the study. An experienced research clinician and exposure technician were present throughout the exposure, with the same researcher and nursing support present during the rest of the protocol.

In the exposure laboratory, participants wore a face mask through which nanoparticles could be delivered by inhalation. Volunteers were asked to cycle at the workload required to increase respiratory rate to 25 l min^−1^ m^−2^ (pre-determined by exercise testing at the screening visit) and rest alternately for 15 min periods across the 2 h exposure. After exposure, the subject returned to the Clinical Research Facility for assessment of biological parameters.

Vital signs, lung function and blood collected pre-exposure (*t* = 0 h), were repeated at *t* = 2.25, 4 and 6 h (that is, 15 min, 2 h and 4 h after exposure). For ease of reading, the 2.25 h time point is referred to as *t* = 2 h throughout). The ex vivo model of deep arterial injury was performed at 1–1.5 h post-exposure, and forearm plethsymography performed at 2–4 h post-exposure (see below). A light lunch was provided that was identical for all volunteers and all study visits. As an additional safety measure, a shortened protocol (without the ex vivo thrombosis assay, forearm plethysmography or 4 h measurements) was performed for the first exposure of each group. The study visits for the subsequent volunteers with the full protocol were scheduled only after it was confirmed that there were no adverse events and no marked changes in blood biomarkers. Volunteers were compensated for their time and travel expenses, which was approved by the ethics committee.

### Lung function and vital signs

The participants were asked to rest in a sitting position for 15 min before measurement of vital signs and lung function. Lung function was measured by spirometry (Vitalograph Alpha III), with the optimal breathing techniques demonstrated at the screening visit. FEV_1_ and FVC were then measured, and a mean of two closely concurring consecutive runs were used. The participants were allowed to rest for a further 5 min before measurement of blood pressure and heart rate by sphygmomanometry.

### Vascular function

The clinical protocol was designed to include measurement of vascular function in response to vasodilator drugs by venous occlusion plethysmography^19^ between *t* = 4 h and *t* = 6 h. However, due to technical and staffing difficulties we were unable to obtain reliable data from sufficient volunteers to make meaningful conclusions, thus the data were omitted. Further details of the technique can be found in the Supplementary Information.

### Blood biomarkers

Blood was sampled before nanoparticle exposure (*t* = 0 h) and at 2, 4 and 6 h. A 17-gauge cannula was inserted into a large antecubital vein of both arms, and flushed with sterile saline. First, 1 ml of blood was discarded and approximately 27 ml was then collected for analysis. EDTA-treated blood was used for measurements of blood cell differentials, citrate-treated blood was used for coagulation markers (activated partial thromboplastin time, prothrombin time, fibrinogen) and clotted blood was used to collect serum for C-reactive protein (CRP) and cytokines (IL-6, TNF-α). Blood measurements were performed by the Clinical Biochemistry Unit at the NHS Royal Infirmary of Edinburgh by the standard methodology. Cytokines were measured using enzyme-linked immunosorbent assay (ELISA) (R&D Systems), with limits of detection of 0.022 pg ml^−1^ for TNF-α and 0.031 pg ml^−1^ for IL-6. Subsamples of blood and urine were frozen at −80 °C for biobanking.

### Ex vivo thrombosis

The coagulability of blood was measured ex vivo using a model of thrombosis on deep arterial injury (Fig. 6). We have used this technique extensively in our clinical studies following exposure of volunteers to diesel exhaust^30,48^ and testing of antithrombotic medication^49,50^. Blood was withdrawn from an antecubital vein via a pump set at a flow rate of 10 ml min^−1^. The first 5 ml of blood was discarded before the cannula was connected using non-coagulation tubing (Masterflex Tygon, Cole Parmer) to three sequential cylindrical perfusion chambers maintained at 37 °C in a water bath. Strips of porcine aorta (Pel-freez) were prepared by carefully removing the intima and a thin layer of media to act as a thrombogenic substrate, and mounted in the chamber according to the physiological direction of blood flow. The rheological conditions in the first chamber simulated those of patent coronary arteries (low-shear rate, ~212 s^−1^), whereas those in the second and third chambers simulate those of mildly stenosed coronary arteries (high-shear rate, ~1,690 s^−1^). The model thus acts as one of deep coronary arterial injury. Each chamber run lasted for 5 min after which saline was perfused over the strip to remove non-adherent blood. The porcine strips with thrombus attached were removed and fixed in 4% paraformaldehyde. Strips were cut into eight cross-sections, wax-embedded, histologically sectioned and endogenous hydrogen peroxide activity blocked with 3% hydrogen peroxide solution. Sections were then incubated at room temperature for 1 h with polyclonal rabbit anti-human fibrin(ogen) antibody (1.2 μg ml^−1^; Cat. No. A0080, Dako) and monoclonal mouse anti-human CD61 antibody (1.28 μg ml^−1^; Cat. No. M0753, Dako). Antigen visualization was performed using a Bond Polymer refine detection kit (Leica Microsystems GmbH) and treatment with 3,3′-diaminobenzidine substrate chromogen (66 mM, Dako). Finally, sections were counterstained with haematoxylin followed by direct red 80 (0.1% sirius red). A semi-automated slide scanner (Axioscan Z1, Zeiss) and image analysis software (QuPath 0.2.3)^51^ were used by a blinded researcher to quantify thrombus area. High-resolution classifiers based on colour were established to detect total thrombus area.

### High-fidelity nanoproteomics analysis of plasma samples

#### Preparation of liposomal nanoparticles and enrichment of plasma proteins

Hyrogenated soybean phosphatidylcholine (HSPC): Cholesterol (Chol):- 1,2-distearoyl-sn-glycero-3-phosphoethanolamine-polyethylene glycol2000 (DSPE-PEG2000) were prepared by thin lipid film hydration followed by extrusion, as previously described^52^. Lipids were dissolved in a 1:1 volume ratio of chloroform:methanol and evaporated under a vacuum. The lipid films were hydrated with ammonium sulfate to produce large multilammer liposomes. Small unilamellar liposomes were produced by extrusion through polycarbonate and extrusion filters (Whatman) using a mini-Extruder (Avanti Polar Lipids).

Liposomes and human plasma were incubated in an orbital shaker, and protein-coated liposomes were separated from excess plasma proteins following a previously described method^53^ using a two-step purification protocol that included size exclusion chromatography and membrane ultrafiltration. Bound proteins were mixed with S-trap lysis buffer containing 5% SDS triethylammonium bicarbonate to allow protein solubilization. Samples were reduced with dithiothreitol, and alkylated with iodoacetamide and dithiothreitol. Protein lysates were mixed with phosphoric acid and S-trap binding buffer to trap proteins in the columns, then digested with trypsin. Peptide samples were extracted and then desalted using oligo R3 beads. Samples were analysed by liquid chromatography mass spectrometry/mass spectrometry using an UltiMate 3000 Rapid Separation lipid chromatography platform (Dionex Corporation) coupled to a Q Exactive Hybrid Quadrupole-Orbitrap mass spectrometer (Thermo Fisher Scientific). The data analysis of the peptide samples is outlined in the Supplementary Information.

### Eicosanoids and related bioactive lipid mediators

Targeted lipidomic analysis was undertaken using a panel of >50 eicosanoids that included prostaglandins (PGD_2_, PGE_2_, PGF_2α_, 13,14-dihydro-15-keto-PGD_2_, 13,14-dihydro-15-keto-PGE_2_, 11-beta-PGF_2α_, 6-keto-PGF_1α_, 15-deoxy-∆^12,14^-PGD_2_, 15-deoxy-∆^12,14^-PGJ_2_); thromboxanes (TxB_2_, 11-dehydro-TxB_2_); hydroxy-eicosatetraenoic acids (5-HETE, 8-HETE, 9-HETE, 11-HETE, 12-HETE, 15-HETE, 20-HETE); leukotrienes (LTB_4_, 20-carboxy-LTB_4_); epoxy-eicosatrienoic acids (5,6-EET, 8,9-EET, 11,12-EET, 14,15-EET; 5-OxoETE, 15-OxoETE); dihydroxy-eicosatrienoic acids (5,6-DHET, 8,9-DHET, 11,12-DHET, 14,15-DHET), hydroxy-eicosapentaenoics acids (5-HEPE, 15-HEPE), octadecadienoic acids (9-HODE; 13-HODE; 9-Oxo-ODE, 13-Oxo-ODE), epoxyoctadecamonoenoic acids (9,10-EpOME, 12,13-EpOME), pro-resolving mediators (lipoxin A_4_ - LXA_4_ and resolvins, RvD1, RvD2); isoprostanes (8-iso-PGF_2α_) and fatty acids (arachidonic acid, eicosapentaenoic acid (EPA), docosahexaenoic acid (DHA) and its metabolites, 7-HDHA; 14-HDHA; 17-HDHA; 10,17-DiHDHA).

Plasma was prepared from EDTA-treated blood. The following internal standards were used: PGE_2_-d_4_, 15-HETE-d_8_, LTB4-d_4_, 14,15- EET-d_11_, 14,15-dHET-d_11_, 9,10-EpOME-d_4_, 9,10-DiHOME-d_4_, RvD2-d_5_, EPA-d_5_ and 8-iso-PGF_2α_-d_4_ (Cayman Chemical). See the Supplementary Information for details of the processing of the samples. Eicosanoids were separated on a Hypersil GOLD C18 column (Thermo) using a Shimadzu Nexera-X2 ultra high performance liquid chromatography system. The effluent was directed into an IonTurbo source of a Sciex QTRAP 6500 mass spectrometer operated in negative-ion mode using multiple reaction monitoring. Eicosanoids were identified on the basis of their characteristic precursor/product ion pair transitions and matching retention time with authentic standards. Data were acquired and analysed using Sciex Analyst^54^ software v1.6. Concentrations of eicosanoids were determined by comparison to a calibration curve run in parallel for each compound and adjusted for recovery by reference to amounts of the appropriate internal standards.

### General data and statistical analysis

Data were analysed using Excel 2010^55^, R 3.2.2 (ref. ^56^) and Prism 9.3 (ref. ^57^). Data in Table 1 and Extended Data Tables 1–6 are presented as mean ± s.d., unless otherwise indicated. Continuous data are presented as means and s.d. Statistical significance within groups and between groups was tested using two-way analysis of variance with Tukey’s honest significant difference post hoc test. Parametric assumptions (normal distribution and equal variances) were confirmed using the statistical packages above; where data were not normally distributed a non-parametric alternative (for example, the Kruskal–Wallis test) was used.

### Reporting summary

Further information on research design is available in the Nature Portfolio Reporting Summary linked to this article.

## Online content

Any methods, additional references, Nature Portfolio reporting summaries, source data, extended data, supplementary information, acknowledgements, peer review information; details of author contributions and competing interests; and statements of data and code availability are available at 10.1038/s41565-023-01572-3.

### Supplementary information

Supplementary Information Supplementary Methods; Results and Discussion; References; Table 1 and Figs. 1 and 2.

Reporting Summary

### Source data

Source Data Fig. 3 and Source Data Extended Data Table 3 Statistical source data.

Source Data Fig. 4 Statistical source data.

Source Data Fig. 5, Source Data Extended Data Table 4 and Source Data Extended Data Table 5 Statistical source data.

Source Data Fig. 6 and Source Data Table 1 Statistical source data.

Source Data Extended Data Table 2 Statistical source data.

Source Data Extended Data Table 6 and Source Data Extended Data Fig. 2 Statistical source data.

## Figures and Tables

**Extended Data Table 1:**
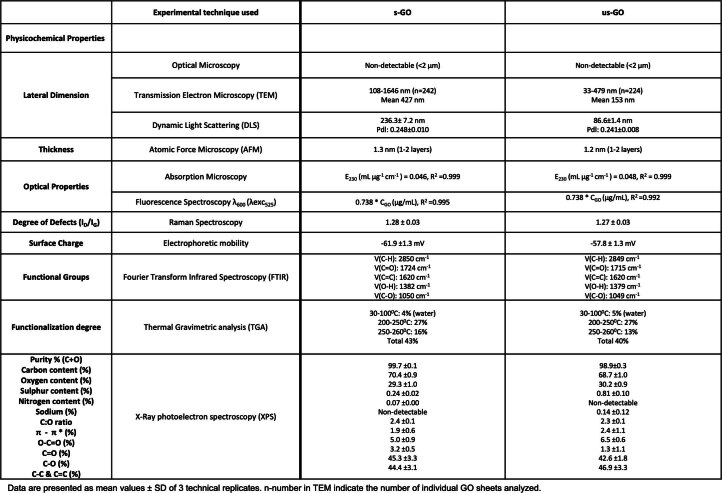
Summary of the physicochemical properties of s-GO and us-GO

**Extended Data Table 2:**
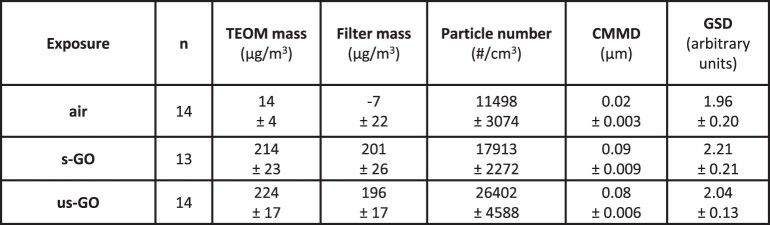
Particle characteristics in the exposure aerosol

Source data

**Extended Data Table 3:**
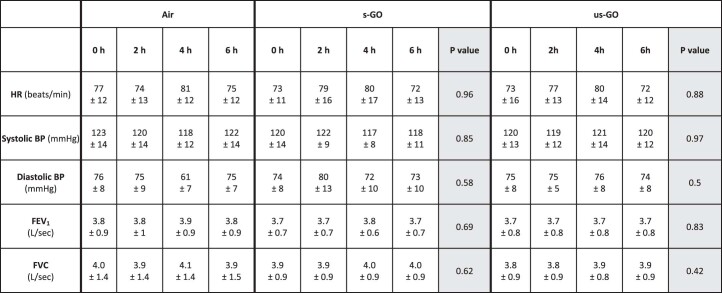
Cardiovascular vital indicators and lung function

Source data

**Extended Data Table 4:**
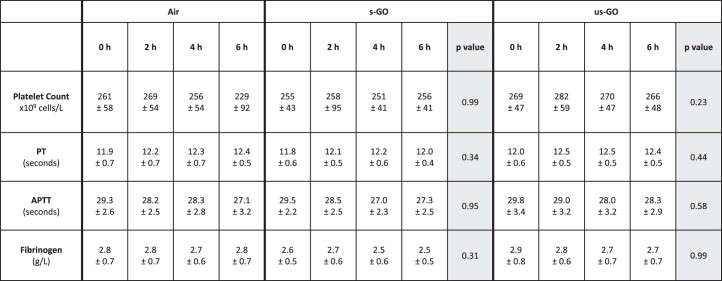
Platelet counts and coagulation markers

Source data

**Extended Data Table 5:**
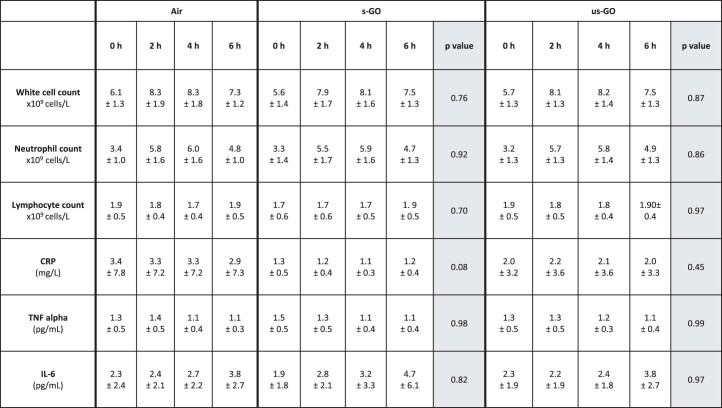
Blood inflammatory cell counts and biomarkers

Source data

**Extended Data Table 6:**
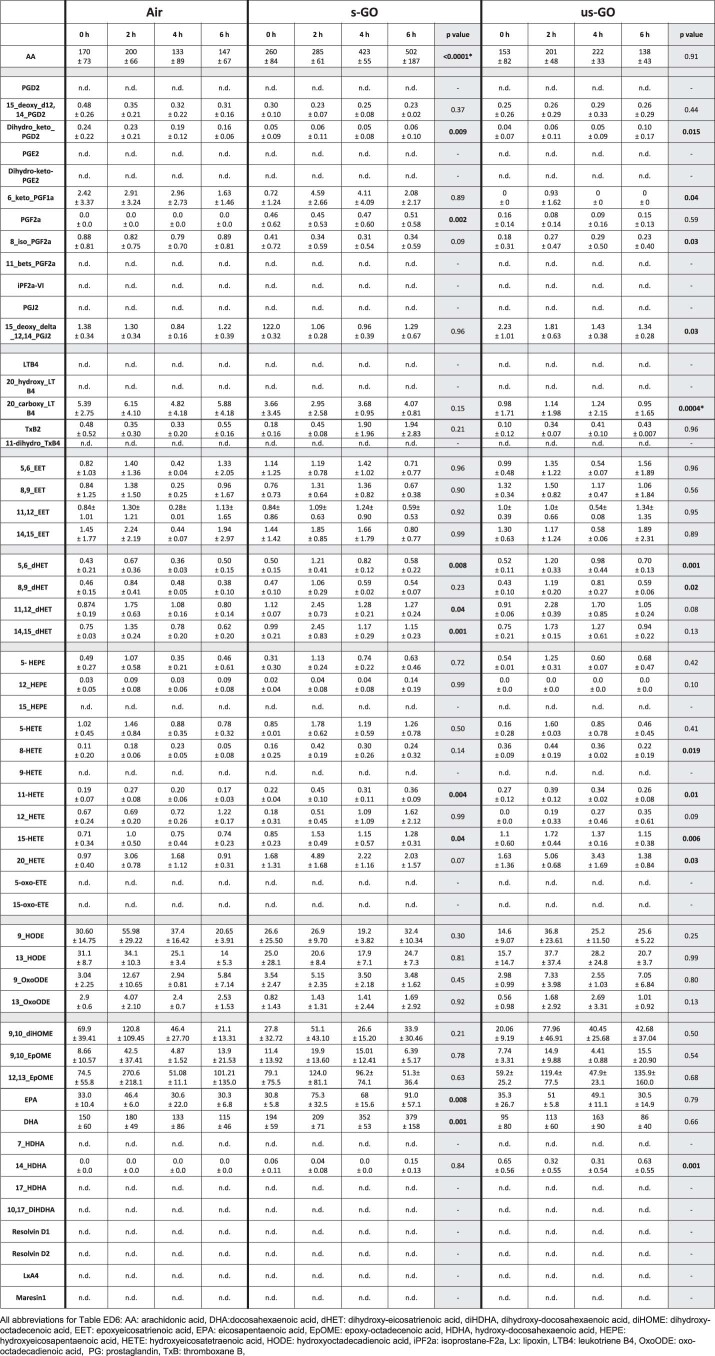
Eicosanoid measurements in blood

Source data

## Data Availability

Data are available through the University of Edinburgh online data repository at 10.7488/ds/7545. Source data are provided with this paper. Correspondence and requests for materials should be addressed to the cooresponding authors M.R.M. or K.K.
