# Supplementary material for: First-in-human controlled inhalation of thin graphene oxide nanosheets to study acute cardiorespiratory responses
Source: Nat Nanotechnol. 2024 Feb 16;19(5):705–14. doi: 10.1038/s41565-023-01572-3 (PMC11106005; doi:10.1038/s41565-023-01572-3)
Supplement: Supplementary file 1 — Supplementary Methods; Results and Discussion; References; Table 1 and Figs. 1 and 2. [file 41565_2023_1572_MOESM1_ESM.pdf]

nature nanotechnology

Article https://doi.org/10.1038/s41565-023-01572-3

First-in-human controlled inhalation of thin graphene oxide nanosheets to study acute cardiorespiratory responses

In the format provided by the authors and unedited

Andrews & Joshi et al.

First-in-human controlled inhalation of thin graphene oxide nanosheets to study acute cardiorespiratory responses

SUPPLEMENTARY MATERIAL

Contents

	Page
**Methods in Full**	**2**
**Supplementary Results and Discussion**	**13**
**Supplementary References**	**17**
**SUPPLEMENTARY TABLE S1.** Volunteer inclusion and exclusion criteria	**20**
**SUPPLEMENTARY FIGURE S1.** Particle exposure characteristics	**21**
**SUPPLEMENTARY FIGURE S2.** Schematic of the mobile exposure laboratory	**22**

Methods in Full

Graphene oxide synthesis

Aqueous dispersions of s-graphene oxide and us-graphene oxide were prepared as described in our previous studies[1,2] by a modified Hummers' method coupled with sonication. We used depyrogenised glassware and graphene oxide suspensions were always handled under endotoxin-free conditions. Graphite powder was mixed with sodium nitrate and sulphuric acid by rigorous stirring at low temperature (ice bath), followed by the addition of potassium permanganate. Water-for-injections was added dropwise to the reaction volume, while carefully monitoring the temperature rise. The mixture was stirred for 30 min at 98°C (oil bath), before stopping the reaction with hydrogen peroxide and leaving for 1 hour. The dispersion was subjected to a series of washes with water-for-injections in order to neutralise the pH, remove the impurities and separate the graphene oxide from the graphitic residues. On the last two washing steps, graphene oxide was exfoliated by vortexing and solubilised in with warm water-for-injections from the orange gel layer which formed at the top of the graphite oxide. Any graphitic residues still present in the dispersion were removed by an additional centrifugation step at 24-h post-reaction. Size reduction to small and ultra-small flakes was carried out by sonication for 5 min and 4-h, respectively.

Characterisation of graphene oxide nanosheets

Graphene oxide was comprehensively characterised (***Main Manuscript Figure 1, Extended Data Table ED1, Figure ED1***) by the following methods:

Atomic force microscopy (AFM). A multimode atomic force microscope (Bruker, UK) was used in tapping mode, using Otespa-R3 probes (Bruker, UK). Samples were prepared on poly-L-lysine 0.01% (Sigma Aldrich P4707) coated mica substrates, by drop-casting a volume of 20 µL of 100 µg/mL graphene oxide dilution in Milli-Q water for 1 min, followed by a washing step with 1 mL Milli-Q water and drying overnight in a drying cabinet (37°C). Scanning parameters were set as follows: 1 Hz scanning rate, 250 mV amplitude set-point, 512 lines per scan, an integral gain of 1 and a proportional gain of 5. Images were processed with the Bruker Nanoscope Analysis software-Version 1.4; the lateral size of the graphene oxide flakes was manually measured by determining the longest Feret diameter in each flake.

Transmission electron microscopy (TEM). TEM analysis was performed on an FEI Tecnai 12 BioTwin equipment (FEI, Eindhoven, NL) with the electron beam set to 100 kV. The samples were prepared on 300-mesh carbon-coated copper grids at room temperature and in a clean environment. A volume of 20 µL of graphene oxide dispersion was drop-casted on the grid and the excess was removed after 1 min with filter paper, leaving a thin layer of suspension to fully dry. A small drop was casted and left to dry at the edge of the grid. Images were captured with an AMT digital camera (Gatan, UK). The raw data

were further processed using ImageJ; the lateral size of the graphene oxide flakes was manually measured by determining the longest Feret diameter in each flake, *n* being the total number of flakes analyzed.

Hydrodynamic diameter and surface charge (zeta potential) measurements. The hydrodynamic diameter and the zeta potential values of graphene oxide suspensions in Milli-Q water were measured with a ZetaSizer Nano ZS instrument (Malvern, UK). The results are reported as the average ± standard deviation of three measurements per sample.

UV-visible spectroscopy (UV-vis). Spectra of graphene oxide dilutions in Milli-Q water with concentrations ranging from 2.5 to 20 μg/mL were acquired using a Cary 50 Bio UV-vis spectrophotometer (Varian Inc., Agilent Technologies, UK). Measurements were performed at room temperature in a quartz cuvette (1 mL volume, 1 cm path length). Milli-Q water was used as a blank.

Fluorescence spectroscopy. Different concentrations of graphene oxide dispersions (25–200 μg/mL) were measured with a Cary Eclipse fluorescence spectrophotometer (Varian Inc., Agilent Technologies, UK). Spectra were acquired at room temperature, with λ_{exc} set to 525 nm. Milli-Q water was used as a blank.

Raman spectroscopy. Measurements were recorded by a DXR micro-Raman spectrometer (Thermo Fisher Scientific, UK) equipped with a 633 nm laser set to 0.4 mW. Calibration was performed on a polystyrene standard, the chosen objective was 50x, and the pinhole was set to 50 μm. Spectra were then recorded between 500 and 4000 cm^{-1} with a resolution of 2.5 cm^{-1}. All spectra were processed by background subtraction and normalisation by the G band intensity using OriginPro 8.5.1 software.

Fourier transform infrared spectroscopy (FTIR). Fourier transform infrared spectra were obtained with a Tensor 27 spectrometer (Bruker, UK), equipped with a 3000 Series High Stability Temperature Controller with RS232 Control (Specac, UK) and a MKII Golden Gate Single Reflection ATR system (Specac, UK) for measurements in ATR mode. The bottom plate of the Golden Gate ATR system was pre-heated at 60°C to allow the complete evaporation of water from the drops (typically 20 μL) of the original graphene oxide dispersions. Approximately 3 min after depositing the dispersions on the plate, the transmittance spectra of graphene oxide were recorded by acquiring 32 scans in the 4000-750 cm^{-1} range, with a resolution of 4 cm^{-1}.

Thermogravimetric analysis (TGA). The oxidation degree of graphene oxide materials was extracted from the degradation patterns measured with a TGA 4000 thermogravimetric analyser (PerkinElmer Ltd, UK). All measurements were carried out on 2 mg lyophilised material, in a nitrogen atmosphere (20 mL/min) at temperatures ranging from 25 to 900°C (10°C/min).

X-ray photoelectron spectroscopy (XPS). XPS measurements of lyophilised graphene oxide samples were analysed using a Thermo Theta Probe XPS spectrometer with a monochromatic Al K- α source of

1486.68 eV. The spectra were acquired with PE of 40 kV, 0.1 eV step size and an average of 20 scans. CasaXPS software (Casa Software Ltd, UK) was used for post-processing of spectra. The contribution of charge injected to insulating samples was corrected by calibrating all peaks according to the adventitious carbon C1s spectral component, set a binding energy of 284.6eV[3]. A Shirley background subtraction was applied to all spectra and Gaussian–Lorentzian (70:30) functions were used for fitting the functional groups, except for the asymmetric C–C and C=C peak, which was fitted using an asymmetric Lorentzian function. The full width half maximum (FWHM) value was constrained between 0.5 and 2 eV for all peaks, except for the π–π*. The following constrain regions were set for the binding energies: 284-285.5 for C-C/C=C, 285.5-286.5 for C-O, 286.8-287.8 for C=O, 288.6-290 for COOH and >290 for π- π*.

Nanoparticle exposure and characterisation

Nanoparticles exposures were performed by the National Institute for Public Health and the Environment (RIVM) in a mobile exposure laboratory positioned outside the main Royal Infirmary of Edinburgh (***Figure S2***), under the supervision of an experienced exposure technician. Stock suspensions of graphene oxide (2 mg/mL for s-graphene oxide; 1.3 mg/mL for us-graphene oxide) were made in sterile distilled water and were free of any bacterial contamination confirmed by absence of bacterial colonies on solid Luria-Bertani agar test and absence of endotoxin (<0.2 EU/µg, level of TNF alpha secreted by bone marrow-derived macrophages exposed to graphene oxide was similar to control untreated cells using previously published method[4]). S-graphene oxide was diluted to 1.3 mg/mL in sterile saline in aseptic conditions, aliquoted and stored at 4°C until use.

Graphene oxide nanosheets (1.3 mg/mL) were aerosolized using a Schlick (Dusen-Schlick, model 970/S Untersiemau, Germany) compressed air nebulizer. Using two syringe pumps (TSE Systems, model 540200, Germany) the suspension was in-line diluted with high-performance liquid chromatography grade water and fed to the Schlick nebulizer. The suspension was transferred to a 5 mL syringe which was placed on the syringe pump and connected to the nebulizer. The compressed pre-heated (60°C) airflow of the Schlick nebulizer was 12l pm. The aerosol was dried in a heated mixing glass tube (90 mm ID, length 550 mm), then diluted with high efficiency particulate air-filtered room air to the desired concentration, humidified to 50-60% relative humidity using an ultrasonic nebulizer (Omron Ultrasonic Nebulizer NE-U12, Japan). The aerosol was fed into a 200 L mixing chamber and delivered to the volunteer by an exposure mask placed over the mouth and nose. Temperature was kept constant throughout and relative humidity of exposure air was 50% maintained using fresh ultrapure water injected into a small side stream using an Omron ultrasonic nebulizer. Graphene oxides were delivered at an exposure concentration between 100 - 300 $\mu g/m^3$, with a target average concentration of 200 $\mu g/m^3$ (***See Figure S1***). This dose range was chosen based on our previous controlled exposure studies with dilute diesel exhaust, which were associated with impairment of a range of cardiovascular

parameters without adverse effects[5,6] and with carbon and gold nanoparticles which did not alter cardiovascular parameters[7,8]. The concentration could be adjusted by altering the speed of the syringe pump delivering the suspension. The real-time mass concentration was measured by a tapered element oscillating microbalance (TEOM; Thermo Scientific, model 1400A, USA) as a guide for changing the speed of the pump. The concentration of the particles in the exposure was monitored and maintained by the exposure technicians.

Particle concentration in the aerosol was taken from the middle of the 200 L mixing chamber by a SS-tube. The particle characteristics measured were: particle mass (TEOM, as well as by gravimetric filter based analyses), particle number (condensation particle counter, TSI Inc. model 3022A CPC, USA), and particle size distribution by a PALAS differential electrical mobility classifier, (U-DEMC model 2200, Germany) and an optical particle sizer (TSI Inc, model 3330, USA). Particle mass was also determined post-exposure by calculating the accumulated mass on pre-weighed telfon filters (Teflo 47 mm, R2PJ047 Pall Corp., USA) taken from the metal tubing close to the volunteer exposure mask, followed by drying and weighing on a microbalance (Sartorius MC5, Germany), and an average taken from 2 duplicate filters.

Ethical Review Statement

This study was designed with rigorous ethical review, with procedures being run by experienced clinicians and nursing support, and performed at a major hospital with the necessary emergency facilities should an adverse event have occurred. The study was performed in accordance with the Declaration of Helsinki, favourable ethical opinion of the University of Edinburgh, NHS Academic and Clinical Central Office for Research and Development (ACCORD), Research Ethics Committee (18-HV-084) and with written informed consent from all participants. The study has been registered on Clinicaltrials.gov (https://www.clinicaltrials.gov/api/gui), reference number: NCT03659864. This research conforms with Nature journals' Author Inclusion and Ethics recommendations.

Participants and eligibility criteria

Fifteen healthy volunteers were recruited by advertising the study by posters and e-mails in the hospital and university campus, as approved by local ethical review. The data from 14 subjects were included as one subject was unable to complete the exposure visits in the time-frame of the study. The target of 15 individuals was based on our previous controlled exposure studies with air pollutants based on changes to vascular reactivity and inflammatory cytokines in the blood, based on diesel exhaust exposure as there is no other controlled exposure study of a two dimensional material for comparisons. A 1-h exposure to diluted diesel exhaust produced an ~32% reduction in forearm bloodflow to 1 nmol/min bradykinin (~16±2 vs ~19±2.5 mL/100 mL tissue/min (±SD) for diesel exhaust vs filtered air control, respectively[9]. A 2-h exposure to diluted diesel exhaust produced a 12.5% increase of plasma TNF-

α (0.99±0.07 vs 0.88±0.007 pg/mL (±SD) for diesel exhaust vs filtered air, respectively[10]. Based on these figures, 12 and 10 volunteers, respectively, would be needed to detect these changes with significant of P<0.05 with an 80% power. Because no other study has tested the effects of an inhaled 2D material, as an additional precautionary step, the decision was taken not to increased group sizes beyond 15 for this study.

Interested volunteers were provided with a participant information sheet which they were asked to read and consider for at least 24 h before agreeing to be involved in the study. For study visits, participants abstained from alcohol for 24 h and from food and caffeine containing beverages for at least 12 h prior to the study visit. Participants were invited for an initial screening visit to ensure that they met the inclusion criteria (***Table S1***). Exclusion criteria included major or traumatic surgery within 12 weeks of screening, a history of and smoking or asthma, occupation with high exposure to air pollution or other inhaled irritant, acute respiratory illness within 3 weeks of enrolment, use of aspirin or anti-inflammatory medication or vitamin and herbal supplements for the week prior to their study visit. Women who were pregnant, lactating or taking contraceptive medication were also excluded from the study. We did not ask participants to wear a facemask outside of the study visits, as low compliance would have added an additional source of variability between participants (the study was run prior to the coronavirus pandemic before mask wearing became common in the UK). Additionally, even occupational facemasks have been shown to vary greatly in their removal of inhaled particles during different modes of activity[11]. Importantly, each volunteer acts as their own control and receives each exposure in a random order, minimising variation from both intrinsic biology and life-style factors.

Study Design

See ***Main Manuscript Figure 2***. A screening visit was used to confirm eligibility criteria with the participant, followed by taking written consent and assignment of a participant code. Height, weight, heart rate, blood pressure and lung function were measured, and a 3-mL blood sample was taken for a full blood cell count. If parameters were within the normal range for young healthy individuals, participants were taken forward to full study days. Additionally, a graded cardio-respiratory exercise stress test on a bicycle ergonometer was performed to determine the workload required to generate a ventilation rate of 25 L/min/m^2.

Two lateral dimensions of GO (maintaining all other physicochemical characteristics almost identical) were selected for the study: 'small' GO (s-GO) and 'ultrasmall' GO (us-GO). Both types of nanosheets have demonstrated neither acute, nor longitudinal adverse effects in our previous pre-clinical (rodent) studies[2], contrary to 'large' GO sheets that were thus excluded from this work as a safety precaution. A double-blind randomised crossover study design was used for the study visits, whereby the order of exposures (filtered air, s-graphene oxide, us-graphene oxide) were randomised. All study visits were organised at least 2 weeks apart to allow a washout period between different exposures. The

volunteer and clinician performing the study were blinded to the identity of the exposure group. All researchers involved with collating and analysing the raw data were blinded to the exposure group, with unblinding occurring only when ready for grouping by exposure.

Prior to exposures (t=0), heart rate, blood pressure and lung function were measured, and blood taken. Participants were asked to empty their bladders and then given a urine container to collect any urine over the course of the study visit. Participants were then taken to the exposure laboratory based at the Royal Infirmary of Edinburgh site for the duration of the study. An experienced research clinician and exposure technician were present throughout the exposure, with the same researcher and nursing support present during the rest of the protocol.

In the exposure laboratory (***Figure S2***), participants wore a face mask through which nanoparticles could be delivered by inhalation. Volunteers were asked to cycle at the workload required to increase respiratory rate to 25 L/min/m^2 (pre-determined by exercise testing at the screening visit) and rest alternatively for 15-min periods across the 2-h exposure. After exposure, the subject returned to the Clinical Research Facility for assessment of biological parameters.

Vital signs, lung function and blood collected pre-exposure (t=0), were repeated at t=2.25, 4 and at 6 h (ie 15 min, 2 h and 4 h after exposure). For ease of reading, the 2.25-h time point is referred to as t=2 throughout the manuscript). The *ex vivo* model of deep arterial injury was performed at 1-1.5 h post exposure, and forearm plethysmography performed at 2-4 h post-exposure (see below). A light lunch was provided that was identical for all volunteers and all study visits. As an additional safety measure, a shortened protocol (without the ex vivo thrombosis and plethysmography or 4-h measurements) was performed for first exposure of each group. The study visits for the subsequent volunteers with the full protocol were scheduled only after it was confirmed that there were no adverse events and no marked changes in blood biomarkers. Volunteers were compensated for their time and travel expenses, which was approved by the ethics committee.

Lung function and vital signs

The participants were asked to rest in a sitting position for 15 min prior to measurement of vital signs and lung function. Lung function was measured by spirometry (Vitalograph Alpha III, UK), with the optimal breathing techniques that were demonstrated at the screening visit. Forced expiratory volume in 1 second (FEV_1) and forced vital capacity (FVC) were then measured, and a mean of two closely concurring consecutive runs were used. The participants were allowed to rest for a further 5 min, prior to measurement of blood pressure and heart rate by sphygmomanometry.

Vascular function

The clinical protocol was designed to include measurement of vascular function by venous occlusion plethysmography between t =4 and t=6 h. However, due to technical and staffing difficulties we were unable to obtain reliable data from sufficient volunteers to make meaningful conclusions, thus the data was omitted. Data analysis was performed after the collection of all study visits, thus the technique was a part of the protocol for all study visits, and subsequently the methods are outlined below. Blood flow was determined using mercury-in-silastic strain gauges placed around each forearm, as previously described[5]. The brachial artery of the non-dominant arm was cannulated with a 27-standard wire gauge steel needle under local anaesthetic. After a 30-minute baseline saline infusion, acetylcholine (endothelium-dependent vasodilator) at 5, 10, and 20 µg/min or sodium nitroprusside (an endothelium-independent vasodilator) at 2, 4, and 8 µg/min were infused at a rate of 1 mL/min for 6 minutes at each dose. Vasodilators were obtained at clinical grade by the Royal Infirmary of Edinburgh Pharmacy. The two vasodilators were separated by 20-minute saline infusions and given in a randomized order, with the researcher blinded to the drug infusion. Expansion of the forearm was measured during venous occlusion and the gradient of the expansion of the forearm was used to determine blood flow, indicating of the ability of arteries to dilate in the presence and absence of vasodilators. Data was recorded on LabChart Reader software (ADInstruments) and exported to Microscoft Excel for analysis.

Blood biomarkers

Blood was sampled before nanoparticle exposure (t=0) and at 2.25, 4 and 6 h. A 17-gauge cannula was inserted into a large antecubital vein of both arms, and flushed with sterile saline. First, 1 mL of blood was discarded and approximately 27 mL was then collected for analysis. EDTA-treated blood was used for measurement blood cell differentials, citrate-treated blood was used for coagulation markers (activated partial thromboplastin time, prothrombin time, fibrinogen) and clotted blood was used to collect serum for C-reactive protein (CRP) and cytokines (IL-6, TNFα). Blood measures were performed by the Clinical Biochemistry Unit at the NHS Royal Infirmary of Edinburgh by standard methodology. Cytokines were measured using ELISA (R&D Systems, UK), with limits of detection of 0.022 pg/mL for TNFα and 0.031 pg/mL for IL-6. Details of -omics analysis of blood factors are described below. Subsamples of blood and urine were frozen at -80°C for biobanking.

***Ex vivo* thrombosis**

The coagulability of blood was measured *ex vivo* using a model of thrombosis on deep arterial injury (***Main Manuscript Figure 6***). We have used this technique extensively in our clinical studies following exposure of volunteers to diesel exhaust[6,12] and testing of antithrombotic medication[13,14]. Blood was withdrawn from an antecubital vein via a pump set at a flow rate of 10 mL/min. The first 5 mL of blood was discarded, before the cannula was connected, using non-coagulation tubing (Masterflex Tygon,

Cole Parmer, UK), to three sequential cylindrical perfusion chambers maintained at 37°C in a water bath. Strips of porcine aorta (Pel-freez, USA) were prepared by carefully removing the intima and a thin layer of media to act as a thrombogenic substrate, and mounted in the chamber according to physiological direction of blood flow. The rheological conditions in the first chamber simulate those of patent coronary arteries (low-shear rate, ~212/s), whereas those in the second and third chambers simulate those of mildly stenosed coronary arteries (high-shear rate, ~1690/s). The model thus acts as one of deep coronary arterial injury. Each chamber run lasted for 5 min after which saline was perfused over the strip to remove non-adherent blood. The porcine strips with thrombus attached were removed and fixed in 4% paraformaldehyde. Strips were cut into 8 cross-sections, wax-embedded, histologically sectioned and endogenous peroxidase activity was blocked using 3% hydrogen peroxide solution (Leica Microsystems GmbH, Wetzlar, Germany) for 5 min. Sections were then incubated at room temperature for 1-h with polyclonal rabbit anti-human fibrin(ogen) antibody (1.2 µg/mL, Dako, Glostrup, Denmark; Cat. No. A0080) and monoclonal mouse anti-human CD61 antibody (1.28 µg/mL, Dako; Cat. No. M0753). Antigen visualization was performed using a Bond Polymer refine detection kit (Leica Microsystems GmbH) and treatment with 3,3′-diaminobenzidine substrate chromogen (66 mM, Dako). Finally, sections were counterstained with haematoxylin followed by direct red 80 (0.1% sirius red).

A semi-automated slide scanner (Axioscan Z1; Zeiss, Jena, Germany) and image analysis software (QuPath 0.2.3) were used by a blinded researcher to quantify thrombus area. Digital images of each section were acquired at ×20 magnification. High-resolution classifiers based on colour were established to detect total thrombus area. Two blinded researchers quality assessed images and sections were discarded if there was evidence of poor vascular integrity, thrombosis forming at a disrupted surface layer, or that the thrombus was dislodged from the arterial strip. Strips with less than three sections per arterial strip were discarded.

High-fidelity nano-proteomics analysis of plasma samples.

Preparation of liposomal nanoparticles and enrichment of plasma proteins. HSPC:Chol:DSPE-PEG2000 (56.3:38.2:5.5) liposomes were prepared by thin lipid film hydration followed by extrusion, as previously described[15]. Lipids were dissolved in chloroform:methanol (4:1) and evaporated (150 rotations/min for 1 h under vacuum, 40°C) using a rotary evaporator (Buchi, Switzerland). Lipid films were hydrated at 60°C with ammonium sulphate (250 mM, pH 8.5) to produce large multilammer liposomes. Small unilamellar liposomes were then produced by extrusion through 800 nm and 200 nm polycarbonate filters (Whatman, UK) for 10 times each, and then 15 times through 100 nm and 80 nm extrusion filters (Whatman, UK) using a mini-Extruder (Avanti Polar Lipids, USA).

Plasma samples used were those obtained at the beginning (t=0) and the end (t=6) of the protocol, with 76 samples analysed in total: air (n=27), s-graphene oxide (n=23) and us-graphene oxide (n=26). Liposomes (180 µL) and human plasma (820 µL) were incubated an orbital shaker

(ThermoFisher, MaxQ™ 4450 Benchtop Orbital Shaker) for 10 min (37°C, 8150*g*). Protein-coated liposomes were separated from excess plasma proteins following a previously described[16]. two-step purification protocol that included size exclusion chromatography and membrane ultrafiltration. Proteins bound to the liposome nanoparticles were quantified by BCA Protein assay kit according to the manufacturer's instructions.

Nanoparticle-bound proteins (10 μg) were mixed with lysis buffer (10 μL) containing 5% SDS, triethylammonium bicarbonate (TEAB, 50 mM,pH 7.5) to allow protein solubilisation. Samples were reduced with dithiothreitol (5 mM), alkylated with iodoacetamide (15 mM) and dithiothreitol (5 nm) added again to quench the alkylation reaction. Samples were centrifuged (14,000*g*, 10 min) to collect the protein lysates, then mixed with phosphoric acid (12%) and six-volume equivalents of S-trap binding buffer (90% aqueous methanol with TEAB (100 mM, pH 7.1)). Samples were added to a S-trap column and centrifuged (4000*g*, 2 min) to trap proteins in the columns. Pelleted proteins were washed four times with S-trap binding buffer, then digested with trypsin (0.1 μg/μL, 47°C, 1 h). Peptide samples were extracted using digestion buffer (50 mM TEAB), 0.1% aqueous formic acid and 30 % aqueous acetonitrile containing 0.1 % formic acid. Finally, peptide samples were desalted by oligo R3 beads in 50% acetonitrile, dried using a vacuum centrifuge (Heto Speedvac) and stored at 4°C until analysed. Samples were analysed by liquid chromatography mass spectrometry (LC-MS)/mass spectrometry (MS) using an UltiMate® 3000 Rapid Separation lipid chromatography platform (RSLC, Dionex Corporation, USA) coupled to a Q Exactive™ Hybrid Quadrupole-Orbitrap™ mass spectrometer (Thermo Fisher Scientific, USA).

Data Analysis. To statistically compare the abundance of proteins identified in the liposomal coronas, mass spectrometry peak intensities were analyzed by importation of the DAW files into Progenesis LC-MS software (version 3.0; Nonlinear Dynamics) with automatic feature detection enabled. A representative reference run was selected automatically, to which all other runs were aligned in a pair-wise manner. Automatic processing was selected to run with applied filters for peaks charge state (maximum charge 5) and a protein quantitation method with relative quantitation using Hi-N with N=3 peptides to measurements per protein. The resulting MS/MS peak lists were exported as a single Mascot generic file and loaded onto a local Mascot Server (version 2.3.0; Matrix Science, UK). The spectra were searched against the UniProt database using the following parameters: tryptic enzyme digestion with one missed cleavage allowed, peptide charge of +2 and +3, precursor mass tolerance of 15 mmu, fragment mass tolerance of 8 ppm, oxidation of methionines as variable modifications and carbamidomethyl as fixed modifications, with decoy database search disabled and ESI-QUAD-TOF as the selected instrument. Each search produced an XML file from Mascot and the resulted peptides (XML files) were imported back into Progenesis LC-MS to assign peptides to features. Data were filtered to present a 1% false discovery rate (FDR) and a score above 21 through the 'refine identification' tab of Progenesis QI toolbox.

Targeted analysis of eicosanoids and related bioactive lipid mediators

Targeted lipidomic analysis was undertaken using a panel of >50 eicosanoids that included *prostaglandins* (PGD_2, PGE_2, $PGF_{2\alpha}$, 13,14-dihydro-15-keto-PGD_2, 13,14-dihydro-15-keto-PGE_2, 11-beta-$PGF_{2\alpha}$, 6-keto-$PGF_{1\alpha}$, 15-deoxy-$\Delta^{12,14}$-PGD_2, 15-deoxy-$\Delta^{12,14}$-PGJ_2); *thromboxanes* (TxB_2, 11-dehydro-TxB_2); *hydroxy-eicosatetraenoic acids* (5-HETE, 8-HETE, 9-HETE, 11-HETE, 12-HETE, 15-HETE, 20-HETE); *leukotrienes* (LTB_4, 20-carboxy-LTB_4); *epoxy-eicosatrienoic acids* (5,6-EET, 8,9-EET, 11,12-EET, 14,15-EET; 5-OxoETE, 15-OxoETE); *dihydroxy-eicosatrienoic acids* (5,6-DHET, 8,9-DHET, 11,12-DHET, 14,15-DHET), *hydroxy-eicosapentaenoics acids* (5-HEPE, 15-HEPE), *octadecadienoic acids* (9-HODE; 13-HODE; 9-Oxo-ODE, 13-Oxo-ODE), *epoxyoctadecamonoenoic acids* (9,10-EpOME, 12,13-EpOME), *pro-resolving mediators* (lipoxin A_4 - LXA_4 and resolvins, RvD1, RvD2); *isoprostanes* (8-iso-$PGF_{2\alpha}$) and *fatty acids* (arachidonic acid - AA, eicosapentaenoic acid - EPA, docosahexaenoic acid - DHA and its metabolites, 7-HDHA; 14-HDHA; 17-HDHA; 10,17-DiHDHA).

Plasma was prepared from EDTA-treated blood. One mL ice-cold methanol containing 1 ng internal standards (PGE_2-d_4, 15-HETE-d_8, LTB4-d_4, 14,15- EET-d_{11}, 14,15-dHET-d_{11}, 9,10-EpOME-d_4, 9,10-DiHOME-d_4, RvD2-d_5, EPA-d_5 and 8-iso-$PGF_{2\alpha}$-d_4; Cayman Chemical, USA) was added to 0.5 mL plasma, after which samples were centrifuged to remove precipitated proteins (600*g* for 10 min at 4°C). The supernatant was diluted to <10% methanol content by the addition of 9 mL double distilled (dd)H_2O and acidified to pH 3.5 with 1 M HCl. Each sample was then applied to a solid-phase extraction (SPE) column (Isolute C18, 500 mg/6 mL, Biotage, Sweden), which had been conditioned with 2 × 6 mL methanol, followed by 2 × 6 mL ddH_2O. The column was subsequently washed with a further 6 mL ddH_2O and 2 × 5 mL hexane before elution of the eicosanoids with 2 × 3 mL ethyl acetate. The ethyl acetate fraction was dried under vacuum and then resuspended in 50:50 (v/v) mobile phase A:mobile phase B, where mobile phase A consisted of H_2O:methanol 90:10 (v/v) containing 0.1% (v/v) acetic acid and mobile phase B consisted of methanol containing 0.1% (v/v) acetic acid. All solvents were LC-MS grade (Fisher Scientific, UK).

Eicosanoids were separated on a Hypersil GOLD C18 column (1.9 µm; 100 x 2.1 mm) (Thermo, UK) using a Shimadzu Nexera-X2 UHPLC system. The initial gradient conditions for analysis were 55% mobile phase A -45% mobile phase B. The percentage of mobile phase B was increased from 45% to 60% over 10 min, followed by 60% to 70% over 1 min, a linear increase to 100% between 11-18 min, held for 2 min before re-equilibration to the starting conditions over 5 min. The flow rate was 400 µL/min. The LC effluent was directed into an IonTurbo source of a Sciex QTRAP 6500 mass spectrometer. The instrument was operated in negative ion mode using the multiple reaction monitoring. Eicosanoids were identified on the basis of their characteristic precursor/product ion pair transitions and matching retention time with authentic standards. Data were acquired and analysed using Sciex Analyst software v1.6. Concentrations of eicosanoids were determined by comparison to a calibration curve run

in parallel for each compound and adjusted for recovery by reference to amounts of the appropriate internal standards.

General data and statistical analysis

Data were analysed using Excel 2010 (Microsoft, USA), R 3.2.2 (R Foundation for Statistical Computing, Austria) and Prism 9.3 (Graphpad, USA). Data in table are presented as mean ± standard deviation, unless otherwise indicated. Continuous data are presented as means and standard deviation and statistical significance within groups and between groups were tested using two-way analysis of variance (ANOVA) with Tukey's Honest Significant Difference post-hoc test. Parametric assumptions (normal distribution and equal variances) were confirmed using the statistical packages above; where data was not normally distributed a non-parametric alternative (e.g. Kruskal-Wallis test) was used.

Supplementary Results and Discussion

Participant characteristics

Fourteen healthy non-smoking volunteers between the age of 18-40 years were recruited for this study. Median height was 172 [169-177] cm. Body weight and body mass index measurements were 65.6 [60.7-71.9] kg and 22.8 [21.1-24] kg/m^2, respectively.

Lung function, heart rate and blood pressure

There were no differences between exposure groups for any of the vital statistics or lung function parameters reported in ***Extended Data Table ED3***. Epidemiological studies have demonstrated that exposure to PM in air pollution is associated with decreases in FEV_1 and FVC[17]. However, controlled exposure studies demonstrate that acute exposure to combustion-derived nanoparticles can induce a pulmonary inflammation, although there is inconsistency in the data as to whether this is associated with changes in airway reactivity[18,19]. In a real-life exposure to diesel emissions in an automotive train, ultrafine- and carbon black-rich diesel exhaust reduced lung function (3.6% reduction in FEV_1) compared to a comparative exposure on an electric train.[20]

A review of pre-clinical studies investigating the effect of GO on pulmonary inflammation[21], identified no direct effects on airway reactivity. Studies in rats have found that a 5-h[22] or 5-day[23] inhalation of mg/m^3 concentrations of GO did not induce pulmonary inflammation in rats, nor did a 28-day exposure to graphene nanoplatelets[24]. Toxicological studies in mice with the same GO materials as the current study found that both a single or repeated pulmonary exposure induced a mild and transient pulmonary inflammation[25–27] which resolved more quickly with smaller (nm range) lateral dimension particles. Neither s-GO nor us-GO nanosheets were associated with airway fibrosis across the 28- or 90-day follow-up[25,28]. Two other studies have found a greater pro-inflammatory effect of larger sizes of GO compared to smaller sizes of the same material[29,30]. Thus, it is feasible that GO with greater lateral dimension could have induced inflammation and changes to lung function.

Three-dimensional human bronchial tissue constructs have been used to demonstrate a pro-inflammatory response to GO[31], albeit at concentrations based on long-term exposure. More recently, the impact of some of the GO used in the present study[32] on a functional human lung organoid system (developed from hES cells) further indicated moderate and transient responses of human lung organoids to ultrasmall GO sheets. While the model and associated data set were not fully available when the controlled human inhalation-exposure study was performed, the three dimensional human lung organoid model confirms the results obtained in mice, highlighting the limited response of human lung organoids to ultrasmall GO sheets and the more adverse effects of large micrometric GO sheets (the latter excluded from our present human study). Biomarkers of pulmonary inflammation would be a valuable addition to future human-controlled nanomaterial exposures and potentially allow

extrapolation from murine models to predict the pulmonary effects of longer-term exposure in humans.

Interestingly, a mouse model of asthma has demonstrated that GO can sensitise airway responsiveness to the agonist methacholine. However, there was no direct effect of GO at the time of the challenge, and responses did not align with markers of pulmonary inflammation[33]. Asthmatic individuals were excluded from our study and represent a potentially susceptible group for future investigations.

Platelet numbers and coagulation markers

There were no differences between exposure groups for any of the blood platelet numbers or coagulation markers reported in ***Extended Data Table ED4***. GO can directly interact with blood to induce conformational changes to fibrinogen and activate complement (C3a, C5a) and intrinsic coagulation (prothrombin) pathways[34]. Direct exposure of platelets to GO can activate platelets and increase the occurrence of thrombus in pulmonary vessels after intravenous injection[35]. However, these effects were dependent on the degree of GO surface functionalisation, while other forms of graphene have minimal direct effects on haemolysis, platelet activation, prothrombin time and activated partial thromboplastin time[36]. It should be borne in mind that these experiments were performed with high concentrations of GO (>0.05 mg/mL) that are orders of magnitude higher than what would be expected to translocate to the circulation after inhalation[8,37]. Accordingly, five days inhalation of GO in rats also showed no effect on these measures of coagulation[23].

Markers of inflammation in blood

GO can activate macrophages through internalisation and induction of complex cellular signalling mechanisms, which are dependent on the dimensions of GO. Smaller nanosheets may have a greater capacity to generate intracellular reactive oxygen species, whereas larger GO can induce necrosis and apoptosis through physical interactions with the cell membrane[38]. In the present study, there was no significant difference between exposure groups for any of the blood inflammatory cells counts or inflammatory cell biomarkers reported in ***Extended Data Table ED5.***

Controlled exposure to diesel exhaust emissions induces a mild increase in blood neutrophils, IL-6 and TNFα[10,19,39], although alterations in these biomarkers have not been consistent between studies. Levels of inflammatory cytokines may have been greater at time points after 6-h, although there was inconsistency across markers of inflammation 24-h after diesel exhaust exposure[10]. Markers of oxidative and genotoxic effects have been shown to be increased, and reduced six months after the installation of workplace filters, in a cohort of six workers in a graphene manufacturing facility[40]. The authors note that it was not possible to discriminate whether these effects could be attributed to nanomaterials or other chemical exposures, but these biomarkers may be suitable for biomonitoring

(see also [41]).We have previously demonstrated that spark-generated carbon black particles are unable to induce a systemic inflammatory response in healthy volunteers[7]. This is also in keeping with the available pre-clinical evidence for graphene materials, where pristine graphene did not directly release cytokines from peripheral blood monocytes[36]. Furthermore, inhalation of GO in mice induced only mild increases in circulatory inflammatory cells or cytokines[22,23] at high doses (>3 mg/m^3) that are likely to be associated with lung overload and do not extrapolate to anticipated real-life exposure scenarios in humans[42,43]. Previous studies have demonstrated that GO can induce an acute phase response in the liver of GO instilled mice[44]. The lack of effect of GO on C-reactive protein in the current study suggests that there was insufficient translocation of GO to the liver, or that the purity of GO may minimise the acute phase response, although the profile of the response at later time points remains to be confirmed. Studies making a direct comparison of our high purity materials and commercial sources of GO that are typically less pure and more heterogenous in their size distribution would be valuable.

Targeted lipidomics to identify the effects of graphene oxide on eicosanoids

Eicosanoids represent a group of diverse mediators formed from the polyunsaturated fatty acid, arachidonic acid. Prostaglandins, leukotrienes, hydroxy-eicosatraenoic acids (HETEs) and epoxyeicosatrienoic/dihydroeicosaatrienoic acids (EETs/dHETs), formed via enzymatic activation of arachidonic acid, are recognised to be important mediators in the onset and progression of inflammation. The free radical-mediated peroxidation of arachidonic acid leads to the production of isoprostanes, which are biomarkers of oxidative stress that is a hallmark of particle-induced cellular dysfunction. Several studies have found that exposure to particulate air pollution in China is associated with inceases in a variety of proinflammatory eicosanoid levels in the blood of humans[45–47]. In mice, inhalation of nanoparticle-rich diesel exhaust has been shown to increase several eicosanoids (HETEs, HODEs, isoprostanes) in the lung lining fluid, plasma, liver and intestines; DE caused oxidative stress and dysfunction of anti-oxidant/anti-inflammatory high-density lipoprotein in the same model[48]. Although very few studies have investigated the effect of graphene materials on eicosanoids, graphene nanoplatelets altered arachidonic metabolism in a macrophage cell line at non-cytotoxic concentrations[49], and low levels of GO modified arachidonic acid and eicosanoids in the brains of zebrafish.

Blood from a subset of participants (n=3) was used to collect preliminary data for eicosanoid profiling. Thirty five out of the fifty five eicosanoid species were detected in the plasma of volunteers, with eighteen species showing a significant difference between the graphene oxides and air (***Extended Data Figure ED2 and Table ED6***) prior to correction for multiple testing. Both s-graphene oxide and us-graphene oxide increased levels of several dHETs and HETEs, whereas greater levels of arachidonic acid, eicosapentaenoic acid and DHA were found after exposure to s-graphene oxide, but not us-graphene oxide. However, after adjusting for multiple comparisons, only six eicosanoids were

significantly different from the air group with a $p \leq 0.001$: 14,15_dHET, arachidonic acid (AA) and docosahexaenoic acid (DHA) were greater for s-GO; 10_carboxy_LTB_4, 5,6_dHET and 14_HDHA were greater for us-GO. 14,15-dHET has been reported to impair neutrophil function[50]. Furthermore, dHETs are metabolites of EETs which have a variety of roles in the lung and cardiovascular system, including vasodilatation, and anti-thombotic and anti-inflammatory properties[51]. 14,15-EET has been shown to provide some protection against cigarette smoke-induced lung injury[52]. However, there was no measurable change in 14,15-EET levels in response to GO suggesting that GO did not have overt effects on EET metabolism. Increased arachidonic acid formation would suggest that s-GO stimulates the activation of phospholipase-A_2 via Ca^{2+} mobilisation, increasing the availability of the substrate for other subsequent eicosanoid pathways. Nano-sized air pollution particles have been shown to increase arachidonic acid and downstream eicosanoids in mice, an effect that was accompanied by inflammation in the gastrointestinal tract (which is exposed to particles following mucocillary clearance from the lung)[53]. In the absence of alterations in downstream eicosanoids, neither of these eicosanoids are likely to represent rate-limiting steps, although it is possible that mobilisation of this substrate may have an influence on eicosanoid formation when other pathways are active, e.g. in the presence of a marked inflammatory response. Different mechanisms may be at play for the release of the fatty acids arachidonic acid and DHA as these are substrates leading to the generation of pro-inflammatory eicosanoids and pro-resolving mediators, respectively. 10_carboxy_LTB_4 is a metabolite of the inflammatory mediator LTB_4, although LTB_4 was not detectable in these plasma samples. 5,6-dHET is also a metabolite 5,6-EETs which has been shown to dilate pulmonary blood vessels[54], however, the latter was not affected by GO exposure. 14_HDHA is a pathway marker for the pro-resolving mediator maresin[55], suggesting the increased production of 14_HDHA could represent the initial stages of a counter response to inflammation, although maresin itself was not significant increased by either GO. However, caution is required in drawing conclusions from a small number of volunteers, and given the small number of eicosanoid species that differed between exposures (6 out of 55 lipids in the panel at $p \leq 0.001$), differences in baseline (t=0) levels of eicosanoids, the small magnitude of differences, and the lack of a consistent pattern for specific graphene oxide sizes, we do not feel it is appropriate to speculate further. Nonetheless, the eicosanoid species identified in the present study, and their regulatory pathways, could be included or targeted in future omic-studies exploring the mechanisms by which graphene oxide, or other MNMs, induce downstream effects.

Supplementary References

1. Jasim, D. A., Lozano, N. & Kostarelos, K. Synthesis of few-layered, high-purity graphene oxide sheets from different graphite sources for biology. *2D Mater.* **3**, 014006 (2016).

2. Rodrigues, A. F. *et al.* A blueprint for the synthesis and characterisation of thin graphene oxide with controlled lateral dimensions for biomedicine. *2D Mater.* **5**, 035020 (2018).

3. Biesinger, M. C. Accessing the robustness of adventitious carbon for charge referencing (correction) purposes in XPS analysis: Insights from a multi-user facility data review. *Appl. Surf. Sci.* (2022).

4. Mukherjee, S. P. *et al.* Detection of endotoxin contamination of graphene based materials using the TNF-α expression test and guidelines for endotoxin-free graphene oxide production. *PLoS One* **11**, e0166816 (2016).

5. Mills, N. L. *et al.* Diesel exhaust inhalation causes vascular dysfunction and impaired endogenous fibrinolysis. *Circulation* **112**, 3930–3936 (2005).

6. Lucking, A. J. *et al.* Diesel exhaust inhalation increases thrombus formation in man. *Eur. Heart J.* **29**, 3043–3051 (2008).

7. Mills, N. L. *et al.* Combustion-derived nanoparticulate induces the adverse vascular effects of diesel exhaust inhalation. *Eur. Heart J.* **32**, 2660–2671 (2011).

8. Miller, M. R. *et al.* Inhaled nanoparticles accumulate at sites of vascular disease. *ACS Nano* **11**, 4542–4552 (2017).

9. Mills, N. L. *et al.* Diesel exhaust inhalation causes vascular dysfunction and impaired endogenous fibrinolysis. *Circulation* **112**, 3930–3936 (2005).

10. Törnqvist, H. *et al.* Persistent endothelial dysfunction in humans after diesel exhaust inhalation. *Am. J. Respir. Crit. Care Med.* **176**, 395–400 (2007).

11. Cherrie, J. W. *et al.* Effectiveness of face masks used to protect Beijing residents against particulate air pollution. *Occup. Environ. Med.* **75**, 446–452 (2018).

12. Lucking, A. J. *et al.* Particle traps prevent adverse vascular and prothrombotic effects of diesel engine exhaust inhalation in men. *Circulation* **123**, 1721–1728 (2011).

13. Wilson, S. J. *et al.* Exosite 1 thrombin inhibition with JNJ-64179375 inhibits thrombus formation in a human translational model of thrombosis. *Cardiovasc. Res.* **115**, 669–677 (2019).

14. Meah, M. N. *et al.* Antithrombotic effects of combined PAR (Protease-Activated Receptor)-4 antagonism and factor Xa inhibition. *Arterioscler. Thromb. Vasc. Biol.* **40**, 2678–2685 (2020).

15. Hadjidemetriou, M. *et al.* In vivo biomolecule corona around blood-circulating, clinically used and antibody-targeted lipid bilayer nanoscale vesicles. *ACS Nano* **9**, 8142–8156 (2015).

16. Hadjidemetriou, M. *et al.* Nano-scavengers for blood biomarker discovery in ovarian carcinoma. *Nano Today* **34**, 100901 (2020).

17. Edginton, S., O'Sullivan, D. E., King, W. & Lougheed, M. D. Effect of outdoor particulate air pollution on FEV 1 in healthy adults: a systematic review and meta-analysis. *Occup. Environ. Med.* **76**, 583–591 (2019).

18. Nightingale, J. A. *et al.* Airway inflammation after controlled exposure to diesel exhaust particulates. *Am. J. Respir. Crit. Care Med.* **162**, 161–166 (2000).

19. Holgate, S. T. *et al.* Health effects of acute exposure to air pollution. Part I: Healthy and asthmatic subjects exposed to diesel exhaust. *Res. Rep. Health. Eff. Inst.* 1–30; discussion 51-67 (2003).

20. Andersen, M. H. G. *et al.* Health effects of exposure to diesel exhaust in diesel-powered trains. *Part. Fibre Toxicol.* **16**, 21 (2019).

21. Pelin, M., Sosa, S., Prato, M. & Tubaro, A. Occupational exposure to graphene based nanomaterials: risk assessment. *Nanoscale* **10**, 15894–15903 (2018).

22. Han, S. G. *et al.* Pulmonary responses of Sprague-Dawley rats in single inhalation exposure to graphene

oxide nanomaterials. *Biomed Res. Int.* **2015**, 1–9 (2015).

23. Kim, Y. H. *et al.* Short-term inhalation study of graphene oxide nanoplates. *Nanotoxicology* **12**, 224–238 (2018).

24. Kim, J. K. *et al.* 28-Day inhalation toxicity of graphene nanoplatelets in Sprague-Dawley rats. *Nanotoxicology* **10**, 891–901 (2016).

25. Rodrigues, A. F. *et al.* Size-dependent pulmonary impact of thin graphene oxide sheets in mice: Toward safe-by-design. *Adv. Sci.* **7**, 1903200 (2020).

26. Loret, T. *et al.* Innate but not adaptive immunity regulates lung recovery from chronic exposure to graphene oxide nanosheets. *Adv. Sci.* **9**, 2104559 (2022).

27. de Luna, L. A. V. *et al.* Lung recovery from DNA damage induced by graphene oxide is dependent on size, dose and inflammation profile. *Part. Fibre Toxicol.* **19**, 62 (2022).

28. Vranic, S. *et al.* Live imaging of label-free graphene oxide reveals critical factors causing oxidative-stress-mediated cellular responses. *ACS Nano* **12**, 1373–1389 (2018).

29. Sydlik, S. A., Jhunjhunwala, S., Webber, M. J., Anderson, D. G. & Langer, R. In vivo compatibility of graphene oxide with differing oxidation states. *ACS Nano* **9**, 3866–3874 (2015).

30. Li, R. *et al.* Enhancing the imaging and biosafety of upconversion nanoparticles through phosphonate coating. *ACS Nano* **9**, 3293–3306 (2015).

31. Di Cristo, L. *et al.* Repeated exposure to aerosolized graphene oxide mediates autophagy inhibition and inflammation in a three-dimensional human airway model. *Mater. Today Bio* **6**, 100050 (2020).

32. Issa, R., Lozano, N., Kostarelos, K., Vranic, S. Functioning human lung organoids model pulmonary tissue response from carbon nanomaterial exposures. BioRxiv April 2023 doi:https://doi.org/10.1101/2023.03.30.534957.

33. Shurin, M. R. *et al.* Graphene oxide attenuates Th2-type immune responses, but augments airway remodeling and hyperresponsiveness in a murine model of asthma. *ACS Nano* **8**, 5585–5599 (2014).

34. Feng, R., Yu, Y., Shen, C., Jiao, Y. & Zhou, C. Impact of graphene oxide on the structure and function of important multiple blood components by a dose-dependent pattern. *J. Biomed. Mater. Res. Part A* **103**, 2006–2014 (2015).

35. Singh, S. K. *et al.* Amine-modified graphene: thrombo-protective safer alternative to graphene oxide for biomedical applications. *ACS Nano* **6**, 2731–2740 (2012).

36. Sasidharan, A. *et al.* Hemocompatibility and macrophage response of pristine and functionalized graphene. *Small* **8**, 1251–1263 (2012).

37. Rodrigues, A. F. *et al.* Size-dependent pulmonary impact of thin graphene oxide sheets in mice: toward safe-by-design. *Adv. Sci.* **7**, 1903200 (2020).

38. Dudek, I., Skoda, M., Jarosz, A. & Szukiewicz, D. The molecular influence of graphene and graphene oxide on the immune system under in vitro and in vivo conditions. *Arch. Immunol. Ther. Exp. (Warsz).* **64**, 195–215 (2016).

39. Salvi, S. *et al.* Acute inflammatory responses in the airways and peripheral blood after short-term exposure to diesel exhaust in healthy human volunteers. *Am. J. Respir. Crit. Care Med.* **159**, 702–709 (1999).

40. Cavallo, D. *et al.* A follow-up study on workers involved in the graphene production process after the introduction of exposure mitigation measures: evaluation of genotoxic and oxidative effects. *Nanotoxicology* **16**, 776–790 (2022).

41. Fadeel, B. *et al.* Safety assessment of graphene-based materials: Focus on human health and the environment. *ACS Nano* **12**, 10582–10620 (2018).

42. Lee, J. H. *et al.* Exposure monitoring of graphene nanoplatelets manufacturing workplaces. *Inhal. Toxicol.* **28**, 281–291 (2016).

43. Vaquero, C., Wendelbo, R., Egizabal, A., Gutierrez-Cañas, C. & López de Ipiña, J. Exposure to graphene in a pilot production plant. *J. Phys. Conf. Ser.* **1323**, 012005 (2019).

44. Bengtson, S. *et al.* Differences in inflammation and acute phase response but similar genotoxicity in

mice following pulmonary exposure to graphene oxide and reduced graphene oxide. *PLoS One* **12**, e0178355 (2017).

45. Wang, T. *et al.* Changes in bioactive lipid mediators in response to short-term exposure to ambient air particulate matter: A targeted lipidomic analysis of oxylipin signaling pathways. *Environ. Int.* **147**, 106314 (2021).

46. Wang, T. *et al.* Proinflammatory lipid signals trigger the health effects of air pollution in individuals with prediabetes. *Environ. Pollut.* **290**, 118008 (2021).

47. Du, X. *et al.* Dynamic molecular choreography induced by traffic exposure: A randomized, crossover trial using multi-omics profiling. *J. Hazard. Mater.* **424**, 127359 (2022).

48. Rezaee, M., Behnam, B., Banach, M. & Sahebkar, A. The Yin and Yang of carbon nanomaterials in atherosclerosis. *Biotechnol. Adv.* **36**, 2232–2247 (2018).

49. Adamson, S. X.-F., Wang, R., Wu, W., Cooper, B. & Shannahan, J. Metabolomic insights of macrophage responses to graphene nanoplatelets: Role of scavenger receptor CD36. *PLoS One* **13**, e0207042 (2018).

50. Bergmann, C. B. *et al.* TPPU treatment of burned mice dampens inflammation and generation of bioactive DHET which impairs neutrophil function. *Sci. Rep.* **11**, 16555 (2021).

51. Zordoky, B. N. M. & El-Kadi, A. O. S. Effect of cytochrome P450 polymorphism on arachidonic acid metabolism and their impact on cardiovascular diseases. *Pharmacol. Ther.* **125**, 446–463 (2010).

52. Yu, G. *et al.* 14,15-Epoxyeicosatrienoic acid suppresses cigarette smoke extract-induced apoptosis in lung epithelial cells by inhibiting endoplasmic reticulum stress. *Cell. Physiol. Biochem.* **36**, 474–486 (2015).

53. Li, R. *et al.* Effect of exposure to atmospheric ultrafine particles on production of free fatty acids and lipid metabolites in the mouse small intestine. *Environ. Health Perspect.* **123**, 34–41 (2015).

54. Stephenson, A. H., Sprague, R. S. & Lonigro, A. J. 5,6-Epoxyeicosatrienoic acid reduces increases in pulmonary vascular resistance in the dog. *Am. J. Physiol. Circ. Physiol.* **275**, H100–H109 (1998).

55. Mozurkewich, E. L. *et al.* Pathway markers for pro-resolving lipid mediators in maternal and umbilical cord blood: A secondary analysis of the mothers, omega-3, and mental health study. *Front. Pharmacol.* **07**, (2016).

SUPPLEMENTARY TABLES AND FIGURES

SUPPLEMENTARY TABLE S1. Volunteer inclusion and exclusion criteria.

INCLUSION CRITERIA	EXCLUSION CRITERIA
Male or female between 18-40 years	Smoked tobacco or related products within one year prior to the study
Judged to be in good health based on medical history, physical examination, vital signs and laboratory tests	Asthmatic
Body mass index between 18-35 kg/m2, and body weight between 50-120 kg	Occupation with a high exposure to air pollution or other inhaled irritants
Willing and able to donate blood	Severe or significant medical condition
Have not taken part in other clinical research within the previous 3 months	Use of any regular prescribed medication within 7 days prior to the study
Willing and able to adhere to complete the screening visit and all 3 study visits	Intercurrent illness (for example: viral cold, influenza, chest infection)
No severe or significant medical condition and without intercurrent illness	Use of aspirin or anti-inflammatory medication in the 7 days prior to the study
	Allergy or contraindication to vasodilator drugs (e.g. acetylcholine or sodium nitroprusside)
	Major or traumatic surgery within 12 weeks of screening
	Pregnant of lactating women
	Given blood in the 3 months prior to the study

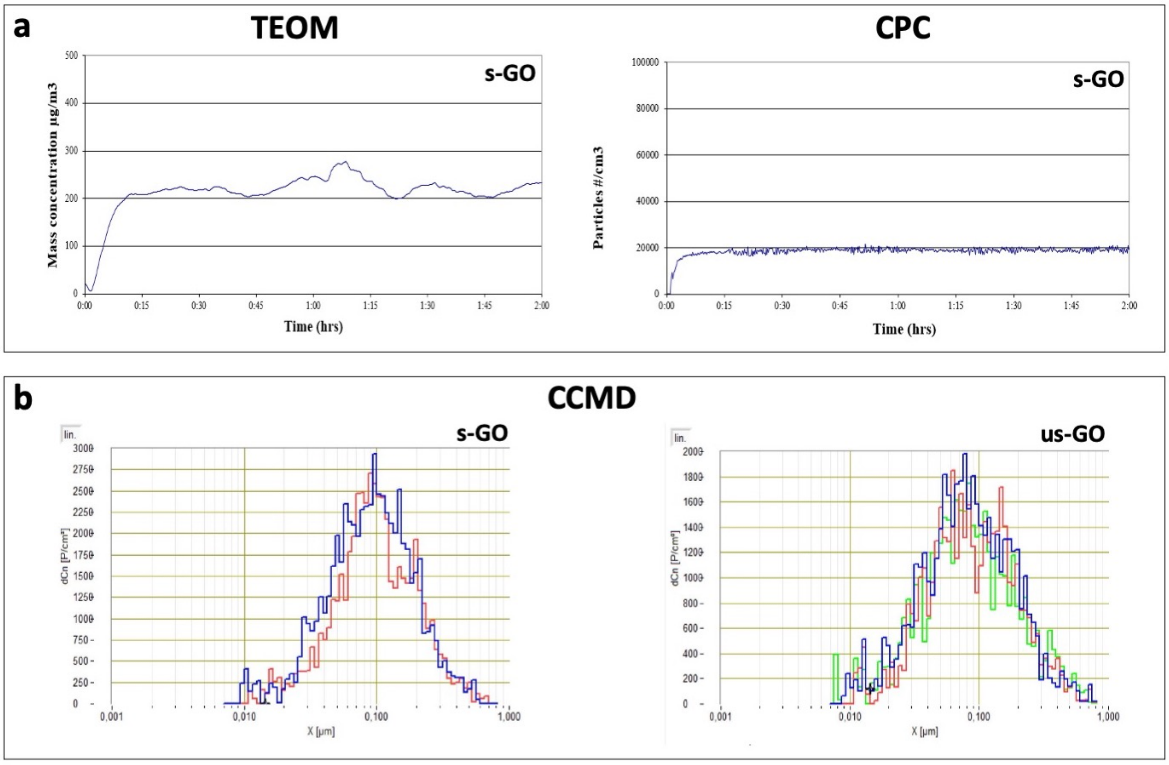

SUPPLEMENTARY FIGURE S1. Particle exposure characteristics. a: Particle mass in exposures measured by tapered element oscillating microbalance (TEOM), condensation particle counter (CPC). **b**: Particle size distribution via differential electrical mobility classifier. CCMD: count median mobility size distribution, s-GO: small graphene oxide, us-GO: ultrasmall graphene oxide.

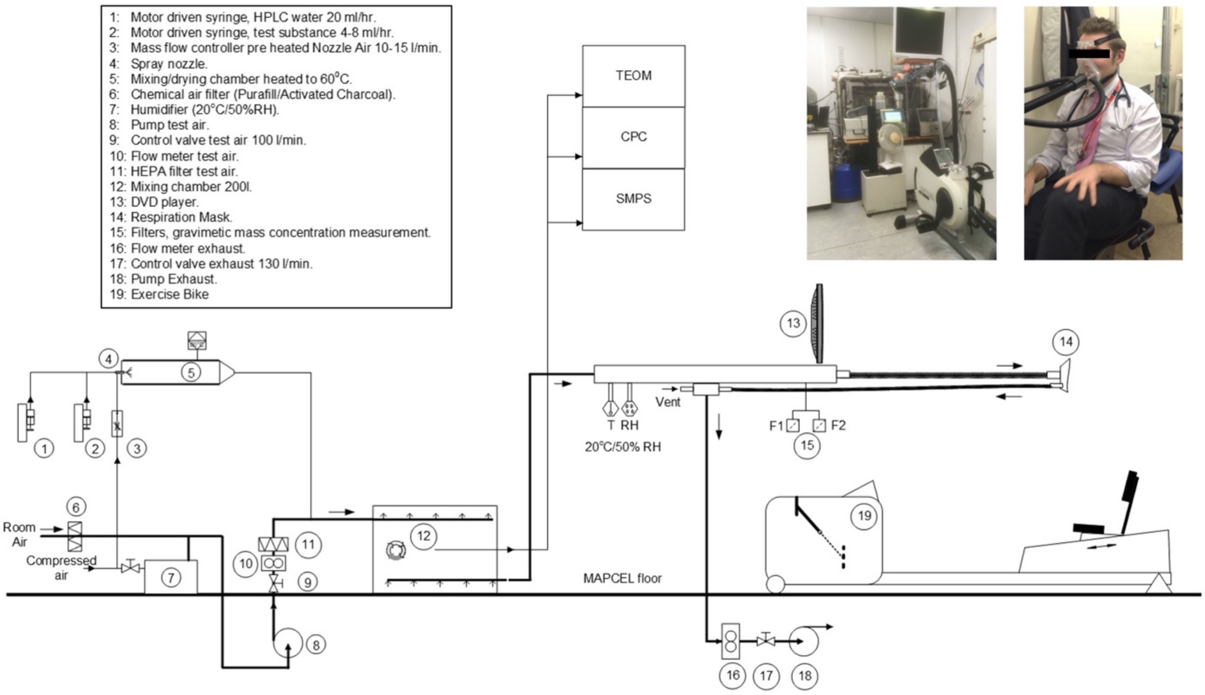

SUPPLEMENTARY FIGURE S2. Schematic of the mobile exposure laboratory. HEPA filter: high efficiency particulate air filter, HPLC water; high-performance liquid chromatography grade water, RH = relative humidity, T: temperature.
