# Supplementary material for: First-in-human controlled inhalation of thin graphene oxide nanosheets to study acute cardiorespiratory responses
Source: Nat Nanotechnol. 2024 Feb 16;19(5):705–14. doi: 10.1038/s41565-023-01572-3 (PMC11106005; doi:10.1038/s41565-023-01572-3)
Supplement: Supplementary file 2 — Reporting Summary [file 41565_2023_1572_MOESM2_ESM.pdf]

nature portfolio

Corresponding author(s): Miller. NNANO-23020355A

Last updated by author(s): Nov 2, 2023

Reporting Summary

Nature Portfolio wishes to improve the reproducibility of the work that we publish. This form provides structure for consistency and transparency in reporting. For further information on Nature Portfolio policies, see our Editorial Policies and the Editorial Policy Checklist.

Statistics

For all statistical analyses, confirm that the following items are present in the figure legend, table legend, main text, or Methods section.

n/a	Confirmed	
☐	☒	The exact sample size (*n*) for each experimental group/condition, given as a discrete number and unit of measurement
☐	☒	A statement on whether measurements were taken from distinct samples or whether the same sample was measured repeatedly
☐	☒	The statistical test(s) used AND whether they are one- or two-sided<br>*Only common tests should be described solely by name; describe more complex techniques in the Methods section.*
☒	☐	A description of all covariates tested
☐	☒	A description of any assumptions or corrections, such as tests of normality and adjustment for multiple comparisons
☐	☒	A full description of the statistical parameters including central tendency (e.g. means) or other basic estimates (e.g. regression coefficient) AND variation (e.g. standard deviation) or associated estimates of uncertainty (e.g. confidence intervals)
☐	☒	For null hypothesis testing, the test statistic (e.g. *F*, *t*, *r*) with confidence intervals, effect sizes, degrees of freedom and *P* value noted<br>*Give P values as exact values whenever suitable.*
☒	☐	For Bayesian analysis, information on the choice of priors and Markov chain Monte Carlo settings
☒	☐	For hierarchical and complex designs, identification of the appropriate level for tests and full reporting of outcomes
☒	☐	Estimates of effect sizes (e.g. Cohen's *d*, Pearson's *r*), indicating how they were calculated

Our web collection on statistics for biologists contains articles on many of the points above.

Software and code

Policy information about availability of computer code

Data collection	no software and code used
Data analysis	no software and code used

For manuscripts utilizing custom algorithms or software that are central to the research but not yet described in published literature, software must be made available to editors and reviewers. We strongly encourage code deposition in a community repository (e.g. GitHub). See the Nature Portfolio guidelines for submitting code & software for further information.

Data

Policy information about availability of data

All manuscripts must include a data availability statement. This statement should provide the following information, where applicable:

- Accession codes, unique identifiers, or web links for publicly available datasets
- A description of any restrictions on data availability
- For clinical datasets or third party data, please ensure that the statement adheres to our policy

The anonymised data collected are available as open data via the University of Edinburgh online data repository, DataShare. [web address and DOI to follow]

Research involving human participants, their data, or biological material

Policy information about studies with human participants or human data. See also policy information about sex, gender (identity/presentation), and sexual orientation and race, ethnicity and racism.

Reporting on sex and gender	Both sexes were recruited as study participants. The study design was repeated measures, thus each individual received all treatments/exposures (in a randomised order), thus all individuals acted as their own control, rather than being treatment groups with different male:female ratio. The group size (n<12) does not allow for analysis of differences between sex or stratification by sex.
Reporting on race, ethnicity, or other socially relevant groupings	Recruitment was carried out independently of race, ethnicity and social grouping. These details were not collected in this study, as is typical of studies of this nature
Population characteristics	The age range recruits was 18-40 years. Body mass index between 18-35 kg/m and body weight was between 50-120 kg. All participants were healthy and not taking regular medication. Full inclusion and exclusion criteria are provided in the manuscript supplement. The study is not powered to address whether the impact on these co-variates
Recruitment	Participants were recruited by poster and E-mail on the hospital/university campus. Details are outlined in the manuscript supplement
Ethics oversight	University of Edinburgh, NHS Academic and Clinical Office for Research and Development (ACCORD), Research Ethics Committee

Note that full information on the approval of the study protocol must also be provided in the manuscript.

Field-specific reporting

Please select the one below that is the best fit for your research. If you are not sure, read the appropriate sections before making your selection.

☒ Life sciences ☐ Behavioural & social sciences ☐ Ecological, evolutionary & environmental sciences

For a reference copy of the document with all sections, see nature.com/documents/nr-reporting-summary-flat.pdf

Life sciences study design

All studies must disclose on these points even when the disclosure is negative.

Sample size	Samples size was determined by power calculations for two key variables based on data from previous controlled exposure studies. These details are now present in the revised manuscript.
Data exclusions	Data points were not excluded from the analysis.
Replication	The study did not set out to reproduce or replicate existing data. To reproduce the current original data would require further controlled exposures in human volunteers which is not possible in the present study
Randomization	All subjects received all treatments/exposures (repeated measures), with the order of the treatment randomised. A sensitivity analysis was performed as part of the manuscript rebuttal, and did not find that the order of the treatment affected key outcomes.
Blinding	Both the participants and the lead clinical researcher making the measurements were blinded to the treatment/exposure. Other researchers analysing the samples were blinded to the exposure until data were ready to group.

Reporting for specific materials, systems and methods

We require information from authors about some types of materials, experimental systems and methods used in many studies. Here, indicate whether each material, system or method listed is relevant to your study. If you are not sure if a list item applies to your research, read the appropriate section before selecting a response.

Materials & experimental systems

n/a	Involved in the study
☐	☒ Antibodies
☒	☐ Eukaryotic cell lines
☒	☐ Palaeontology and archaeology
☒	☐ Animals and other organisms
☐	☒ Clinical data
☒	☐ Dual use research of concern
☒	☐ Plants

Methods

n/a	Involved in the study
☒	☐ ChIP-seq
☒	☐ Flow cytometry
☒	☐ MRI-based neuroimaging

Antibodies

Antibodies used	polyclonal rabbit anti-human fibrin(ogen) antibody (1.2 μg/mL, Dako, Glostrup, Denmark; Cat. No. A0080) and monoclonal mouse anti-human CD61 antibody (1.28 μg/mL, Dako; Cat. No. M0753).
Validation	No specialist validation of antibodies was required - these antibodies were used to provide clear contrast of blood cells from underlying smooth muscle of tissue, rather than assessment of subtle changes in expression

Clinical data

Policy information about clinical studies

All manuscripts should comply with the ICMJE guidelines for publication of clinical research and a completed CONSORT checklist must be included with all submissions.

Clinical trial registration	ClinicalTrials.gov reference number NCT03659864
Study protocol	The study protocol will be made available on the University of Edinburgh's Data Repository alongside the data
Data collection	All primary clinical data and biological specimens were collected on the University of Edinburgh/Royal Infirmary of Edinburgh Site between 3 Oct 2018 to 29 March 2019
Outcomes	Otcomes measured included blood eicosanoids, urine eicosanoids, blood pressure, heart rate, lung function (FEV1), lung function (FVC), coagulation screen, internal normalised ratio, C-reactive protein, tumour necrosis factor, interleukin-6, area of thrombus (low stress), area of thrombus (high stress), forearm blood flow (ACh), forearm blood flow (SNP). These were based and defined on previous controlled exposure studies (majority of clinical parameters) and parallel preclinical studies (eicosanoids) funded by the same grants
